# Targeting TTK signaling axis: chromosomal instability to tumor fitness

**DOI:** 10.1186/s12964-026-03139-w

**Published:** 2026-08-14

**Authors:** Prachi Patidar, Kritika Jangir, Chainsee Saini, Prerna Vats, Bhavika Baweja, Niranjan Kumar, Rashi Soni, Ashok Kumar, Rajeev Nema

**Affiliations:** 1 Department of Biosciences, Manipal University Jaipur, Dehmi Kalan, Jaipur-Ajmer Expressway, Jaipur, Rajasthan 303007 India; 2 Department of Biochemistry, All India Institute of Medical Sciences (AIIMS), Saket Nagar, Bhopal, Madhya Pradesh, 462020 India

**Keywords:** TTK/Mps1 Kinase, SAC, Kinetochore, Microtubule Attachment, CIN, Aneuploidy, Mitotic Catastrophe, Tumor Adaptability, DDR, EMT, Oncogenic Signaling, Small-Molecule Inhibitors and Precision Oncology

## Abstract

Threonine tyrosine kinase (TTK) also known as MonoPolar Spindle 1 (Mps1) is a core regulator of the spindle assembly checkpoint (SAC) that ensures accurate chromosome segregation during mitosis. While its canonical role in safeguarding genomic stability is well established, emerging evidence indicates that TTK is frequently overexpressed across a wide range of cancers, where it supports tumor cell survival under conditions of elevated chromosomal instability (CIN). This paradox highlights TTK's dual role as both a guardian of mitotic fidelity and a facilitator of tumor adaptability. Recent studies have demonstrated that pharmacological inhibition of TTK induces mitotic catastrophe by exacerbating chromosome mis-segregation, thereby selectively targeting cancer cells. Several small-molecule TTK inhibitors have entered clinical evaluation, particularly in combination with chemotherapeutic agents and radiotherapy. However, therapeutic responses remain heterogeneous, and the mechanistic basis of tumor-specific reliance on TTK across different cancer contexts remains poorly understood. This review consolidates current knowledge of TTK function beyond its canonical SAC activity, emphasizing its emerging roles in DNA damage response, epithelial-mesenchymal transition, and oncogenic signaling pathways. In addition, the review highlights critical unresolved questions, including the distinction between kinetochore and non-kinetochore functions, and the quantitative thresholds of TTK activity required for checkpoint maintenance. Finally, current advances in TTK-targeted drug development, challenges related to therapeutic selectivity and resistance, along with rational combination therapies and biomarker-guided therapeutic strategies have been summarized. Addressing these challenges will be critical for translating TTK biology into effective therapeutic interventions and for harnessing its potential in precision oncology.

## Highlights

- TTK serves as a central regulator of the SAC, ensuring accurate chromosome segregation and mitotic fidelity.
- TTK participates in DDR pathways, contributing to checkpoint activation and maintaining genome stability under stress.
- Aberrant overexpression of TTK is associated with CIN, aggressive tumor behavior, and poor clinical outcomes across multiple cancers.
- TTK promotes tumor progression by supporting proliferation, stress adaptation, and survival through the integration of mitotic and non-canonical signaling pathways.
- Therapeutic targeting of TTK, alone or in combination, represents a promising approach to exploit mitotic vulnerabilities in cancer.

## Introduction

Correct chromosome segregation during mitosis is essential for maintaining genomic integrity, and its disruption leads to chromosomal instability (CIN), a hallmark of cancer [1]. To prevent aberrant segregation, eukaryotic cells rely on the spindle assembly checkpoint (SAC), a surveillance mechanism that delays anaphase onset until all chromosomes are properly attached to the mitotic spindle [2]. Disruption of SAC-associated kinases contributes directly to mitotic failure and tumorigenesis [3]. While this checkpoint preserves genomic fidelity in normal cells, cancer cells frequently exhibit elevated CIN, raising a fundamental question: how do tumor cells tolerate persistent mitotic errors while maintaining proliferative capacity? Recent evidence suggests that cancer cells do not simply bypass mitotic checkpoints but instead adaptively rewire them. In this context, Threonine tyrosine kinase (TTK), also known as Mps1, has emerged as a central regulator of mitotic progression. TTK orchestrates SAC activation through phosphorylation-dependent recruitment of checkpoint proteins at kinetochores, thereby preventing premature anaphase onset [4].

Its orthologs are broadly conserved from yeast to higher eukaryotes, highlighting its essential role in mitotic checkpoint control and chromosome segregation fidelity [5–9]. Paradoxically, TTK is often overexpressed in diverse malignancies, where its activity appears to support tumor cell survival despite ongoing chromosomal instability [10]. This dual role of TTK highlights a critical conceptual shift: rather than functioning solely as a guardian of genomic integrity, TTK may act as a regulator of mitotic stress tolerance, enabling cancer cells to balance the detrimental and adaptive consequences of CIN [11]. Consistent with this view, inhibition of TTK disrupts checkpoint signaling and drives catastrophic chromosome mis-segregation, selectively impairing tumor cell viability. These observations have led to the development of small-molecule TTK inhibitors, several of which are currently undergoing clinical evaluation. Despite these advances, key questions remain unresolved, such as, the mechanisms governing TTK activation and spatiotemporal regulation are incompletely understood, particularly with respect to kinetochore versus non-kinetochore functions. Key questions remain regarding the determinants of tumor-specific TTK expression, including the roles of CIN thresholds and compensatory signaling pathways, which are not yet fully defined. Although TTK has been implicated in diverse oncogenic processes, these functions are not equally central to tumor biology. Its canonical role in SAC signaling represents the primary molecular function that preserves chromosome segregation fidelity. This review provides a comprehensive analysis of TTK function in mitosis and cancer, with particular emphasis on its emerging roles beyond canonical SAC signaling. Current therapeutic strategies targeting TTK are critically examined, along with the challenges associated with clinical translation, including resistance mechanisms and the need for predictive biomarkers. A unifying framework is proposed in which TTK functions as a key modulator of mitotic stress tolerance, highlighting its potential as a target for cancer therapy.

## History and evolutionary conservation of TTK

Originally identified in the mid-1990s through genetic screens in the budding yeast *Saccharomyces cerevisiae*, Mps1 (monopolar spindle 1) was characterized by Weiss and Winey [9] as a critical regulator of spindle pole body (SPB) duplication and mitotic checkpoint control. Before the characterization of yeast Mps1, the human TTK gene had been identified in 1992 as a novel protein kinase associated with cell proliferation, providing an early independent description of the mammalian ortholog [12]. Furthermore, the loss-of-function studies showed that Mps1 deficiency leads to monopolar spindle formation and failure to arrest in mitosis under spindle stress, establishing its role in coordinating centrosome dynamics and checkpoint signaling [9, 13, 14]. Subsequent studies across model organisms revealed two principles that have remained conserved throughout eukaryotic evolution: first, TTK dynamically localizes to kinetochores in response to unattached or improperly attached microtubules, and second, its catalytic activity is essential for generating the inhibitory checkpoint signal that restrains premature anaphase onset [15]. These features position TTK as a key link between kinetochore attachment status and cell-cycle progression.

Phylogenetic analyses show that TTK is conserved across eukaryotes, from yeast to vertebrates. This conservation is strongest within the C-terminal kinase domain, reflecting selective pressure to preserve checkpoint function [16]. In contrast, the N-terminal region is more divergent, consistent with kinetochore targeting and regulatory control [17]. Although the core role of Mps1/TTK in kinetochore surveillance and SAC signaling is broadly conserved, its regulatory mechanism and additional cellular functions have diversified across species. This modular architecture enables species-specific regulation while preserving core checkpoint signaling. Collectively, the evolutionary trajectory of MPS1 reflects a finely tuned balance between stringent conservation of its catalytic checkpoint-activating core and diversification of regulatory modules that enable adaptive control of kinetochore signaling. This architecture has allowed SAC surveillance to remain robust across eukaryotic organisms of increasing cellular and genomic complexity. Importantly, the strong evolutionary conservation of TTK, particularly within its catalytic kinase domain, underscores its functional indispensability and therapeutic relevance. This conserved architecture provides a foundation for developing inhibitors that exploit tumor-specific dependence on TTK-mediated mitotic control.

## Structural basis of TTK

**Fig. 1:**
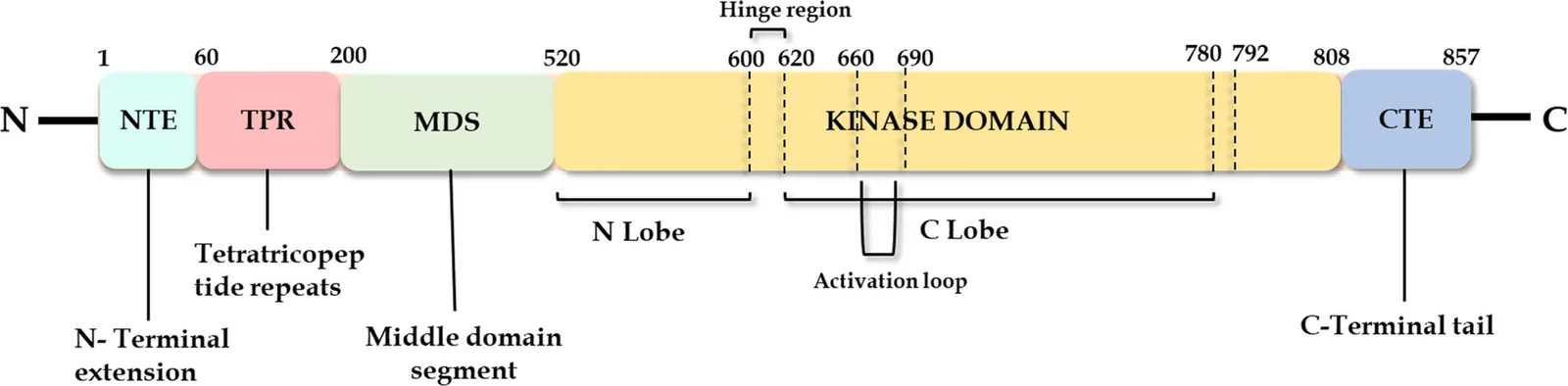
Structural organization and domain architecture of the TTK kinase. Schematic representation of the linear domain organization of the TTK protein from the N-terminus to the C-terminus. The N-terminal extension (NTE; residues 1–60) is followed by tetratricopeptide repeat (TPR) motifs (residues 60–200), which are implicated in protein–protein interactions and kinetochore recruitment. The central region comprises the middle domain segment (MDS; residues 200–520), which contributes to structural stability and regulatory coordination. The catalytic serine/threonine kinase domain (~ 520–808) occupies the core functional region and is subdivided into an N-lobe and a C-lobe, connected by a hinge region (residues 600–620), which facilitates ATP binding and substrate recognition. Within this domain, the activation loop (residues 660–690) plays a critical role in modulating kinase activity and conformational switching. The protein terminates with a C-terminal extension (CTE; ~ 808–857), which may contribute to substrate specificity and regulatory interactions. Numbers indicate approximate amino acid positions along the TTK sequence

Human TTK, a serine/threonine kinase, is 857 amino acids long, whose modular architecture enables precise coupling between regulatory control, spatial localization, and catalytic activity [12, 18]. Although the full-length structure of TTK has not yet been resolved, extensive structural, biochemical, and computational studies of individual domains, particularly the catalytic core have provided detailed mechanistic insight into kinase activation, conformational dynamics, and inhibitor engagement. Based on sequence annotation and structural analyses, TTK can be subdivided into five major regions: an N-terminal extension (NTE), a tetratricopeptide repeat (TPR) domain, a central regulatory middle region (MR), also referred to as the middle domain segment (MDS) in structural studies, a bilobed catalytic kinase domain, and a short C-terminal tail [19–21], as discussed below and illustrated in Fig. 1.

### N-terminal regulatory architecture: NTE and TPR domains

The NTE (residues ~ 1–60) is partially intrinsically disordered and primarily confers regulatory flexibility rather than catalytic activity. Positioned upstream of the structured TPR domain, the NTE likely provides conformational adaptability required for spatial regulation and dynamic protein–protein interactions. Although high-resolution structural information for this region is lacking, domain mapping and mutational analyses consistently identify it as the most N-terminal regulatory segment of the protein [22]. Immediately following the NTE, the TPR region (approximately residues ~ 60–200) comprises α-helical tetratricopeptide repeat motifs that form a conserved protein–protein interaction scaffold [23]. While atomic-resolution structures of the isolated TPR domain are not available within existing kinase-domain crystal structures, sequence conservation and structural modeling identify this region as a critical interaction module positioned upstream of the catalytic core [22]. The TPR domain plays a central role in mediating intermolecular interactions required for kinetochore recruitment and regulatory control, rather than enzymatic catalysis [21].

### Central regulatory middle domain segment

The MR/MDS (approx. 200–520 residues) links the N-terminal targeting modules to the catalytic kinase domain and functions as a regulatory interface. A conserved region within the MR contributes to kinetochore association through interaction with NUF2, a component of the NDC80 complex, and can also influence catalytic activity through communication with the kinase domain [24]. This region may also contains multiple phosphorylation sites that modulate kinase activation, conformational stability, and intramolecular communication between regulatory and catalytic elements [25]. The MR/MDS therefore provides an additional regulatory interface that links kinetochore-associated interactions with catalytic regulation and helps integrate signals and post-translational modifications to fine-tune catalytic output.

### Catalytic kinase domain

The most structurally well-defined region of TTK is its catalytic kinase domain (~ 520–808 residues), which adopts the canonical bilobed protein kinase fold [26]. Its N-lobe spans residues ~ 520–600 and is composed primarily of a five stranded β-sheets and the α-helical elements characteristic of the kinase N-lobe, containing the conserved glycine-rich loop essential for ATP coordination [25]. The C-lobe (approximately residues ~ 620–780) is predominantly α-helical and contains the catalytic loop, substrate recognition surfaces, and the activation segment. A key regulatory element within this lobe is the activation loop (approximately residues ~ 660–690), which exhibits marked conformational plasticity. Crystallographic analyses reveal that the activation loop can adopt either an ordered or a disordered conformation depending on its phosphorylation status [27]. In the unphosphorylated kinase, the activation loop can adopt an inhibitory configuration that is associated with displacement of the αC helix, disruption of the catalytic Lys553-Glu571 salt bridge, and misalignment of the regulatory hydrophobic spine, thereby stabilizing an inactive kinase conformation [27]. Autophosphorylation within this loop induces structural rearrangements that stabilize the active kinase conformation by aligning catalytic residues and facilitating efficient substrate engagement. Phosphorylation of Thr676 is particularly important for TTK activation and spindle assembly checkpoint function, although activation of the kinase involves a multistep phosphorylation-dependent process involving additional regulatory sites [25, 27]. Functional and structural studies consistently demonstrate that activation loop phosphorylation is essential for full catalytic activity [28]. Embedded within the activation segment is a conserved DFG motif that governs the transition between inactive and active kinase conformations. Structural repositioning of this motif regulates ATP coordination and catalytic alignment, underscoring its central role in kinase activation dynamics [26]. Comparative analyses further support a model in which phosphorylation-dependent stabilization of the activation loop shifts TTK from an inactive to an active configuration, effectively functioning as a molecular switch that controls catalytic output [27]. In between the N-lobe and C-lobe, the ATP-binding cleft is formed, which is stabilized by a conserved hinge region (approximately residues ~ 600–620), which mediates critical hydrogen-bonding interactions with ATP and small-molecule inhibitors [29]. Structural studies of inhibitor-bound TTK, including complexes with SP600125, demonstrate that the hinge region constitutes the core ligand-binding interface and plays a major role in determining inhibitor specificity and binding affinity [29]. The adjacent gatekeeper residue Met602 also contributes to the architecture of the hydrophobic pocket and influences the binding of structurally diverse TTK inhibitors [30]. These features have made the hinge region and adjoining ATP-binding pocket a focal point for rational inhibitor design.

### C-terminal tail and global architectural integration

Beyond the structured catalytic core, the C-terminal tail (~ 808–857 residues) provides an additional regulatory extension. Although this region is poorly resolved structurally, biochemical and truncation studies suggest that it contributes to protein stability, substrate accessibility, and intramolecular regulation rather than direct catalysis [22]. This structural framework reveals a highly modular architecture in which the N-terminal regulatory domain governs localization and conformational flexibility, while the bilobed kinase domain executes phosphorylation via a tightly regulated, activation-loop–dependent mechanism. Autophosphorylation of the activation segment stabilizes the active state, the hinge region defines ATP and inhibitor interactions, and conformational plasticity within the conserved Asp-Phe-Gly (DFG) motif, which governs transitions between inactive and active configurations. These structural insights not only explain the molecular basis of TTK activation and regulation but also provide a robust framework for rational inhibitor development and therapeutic targeting, particularly in chromosomally unstable cancers that exhibit dependency on TTK-mediated mitotic control [22].

## Architecture-driven recruitment of TTK to Mitotic kinetochore

Recruitment of TTK to mitotic kinetochores is not a passive diffusion-driven event but a structurally encoded process governed by its N-terminal architecture and the molecular organization of the outer kinetochore [31]. Spatial confinement of TTK activity to unattached or improperly attached kinetochores is essential to ensure that checkpoint signalling and error correction are initiated only where required [32]. This localization is primarily mediated by the N-terminal region of TTK, particularly the TPR domain, which functions as a dedicated interaction scaffold rather than a catalytic module [33]. Structural and functional analyses demonstrate that the TPR domain adopts a conserved α-helical fold optimized for protein–protein interactions and is indispensable for kinetochore localization [34]. Deletion or mutation within this N-terminal module results in a profound reduction in kinetochore recruitment and checkpoint competence, establishing this region as the principal targeting interface [33]. Importantly, the adjacent NTE does not operate independently; intramolecular interactions between N-terminal modules modulate exposure of binding surfaces and influence the conformational state of TTK. These interactions are required not only for recruitment but also for proper checkpoint silencing, indicating that the N-terminus integrates localization with regulatory control [35]. Following recruitment mediated by the N-terminal architecture, TTK engages specific kinetochore receptors to stabilize its localization. Biochemical reconstitution and cellular analyses reveal that TTK interacts with Ndc80, a core outer kinetochore microtubule-binding complex [36], through multivalent interactions involving multiple contact points rather than a single high-affinity interface [24]. This multisite interaction strategy enhances binding avidity while preserving reversibility, allowing TTK to accumulate robustly at unattached kinetochores yet disengage efficiently once attachment status changes [37]. TTK dimerization further reinforces this recruitment mechanism. The C-terminal region contributes to oligomerization, increasing the effective local concentration of N-terminal docking modules and stabilizing association with the Ndc80 complex [38]. Dimerization-dependent multisite engagement creates a responsive binding system in which TTK occupancy reflects the kinetochore's attachment state.

Crucially, recruitment is intrinsically coupled to microtubule attachment status through structural competition. The binding interface for TTK on the Ndc80 complex partially overlaps with microtubule-binding surfaces. As microtubule end-on attachments mature, physical engagement of microtubules with Ndc80 sterically and competitively limits TTK association, promoting its displacement from satisfied kinetochores [39]. This competitive exclusion mechanism ensures that TTK is selectively retained at unattached or low-tension kinetochores while being removed upon stable microtubule attachment [32]. Phospho regulation further refines this dynamic cycle. The kinetochore operates as a communication hub integrating kinase and phosphatase activities, and dephosphorylation events contribute to the release of TTK once proper attachment is achieved [40]. Thus, TTK localization is governed by a coordinated network involving N-terminal structural architecture, multivalent Ndc80 engagement, dimerization-dependent avidity, microtubule competition, and phospho regulated turnover. Through this architecture-driven recruitment system, TTK is deployed precisely where mechanical and attachment errors persist. By embedding localization logic into its structural framework, TTK ensures that downstream checkpoint signaling and error-correction mechanisms are activated in a spatially restricted, attachment-sensitive manner, thereby safeguarding mitotic fidelity.

## Canonical role of TTK

Building on its architecture-driven recruitment to mitotic kinetochores, TTK executes its core functions in maintaining mitotic fidelity by coordinating SAC signaling and kinetochore–microtubule error correction. Once localized, TTK acts as a dynamic signaling hub that translates attachment status into checkpoint activation while simultaneously modulating microtubule attachment stability. Through this dual functionality, TTK ensures that chromosome segregation is tightly coupled to proper spindle dynamics, thereby preventing premature anaphase onset and preserving genomic stability.

### TTK in SAC

TTK functions as the central initiating kinase of the SAC, acting as the catalytic engine that links kinetochore attachment status to inhibition of anaphase onset [15]. At the onset of mitosis, when kinetochores are incorrectly attached, TTK is recruited to the outer kinetochore through interactions with the Ndc80 complex. This localization is tightly controlled by microtubule occupancy and represents the earliest decisive step in checkpoint activation [41].

**Fig. 2:**
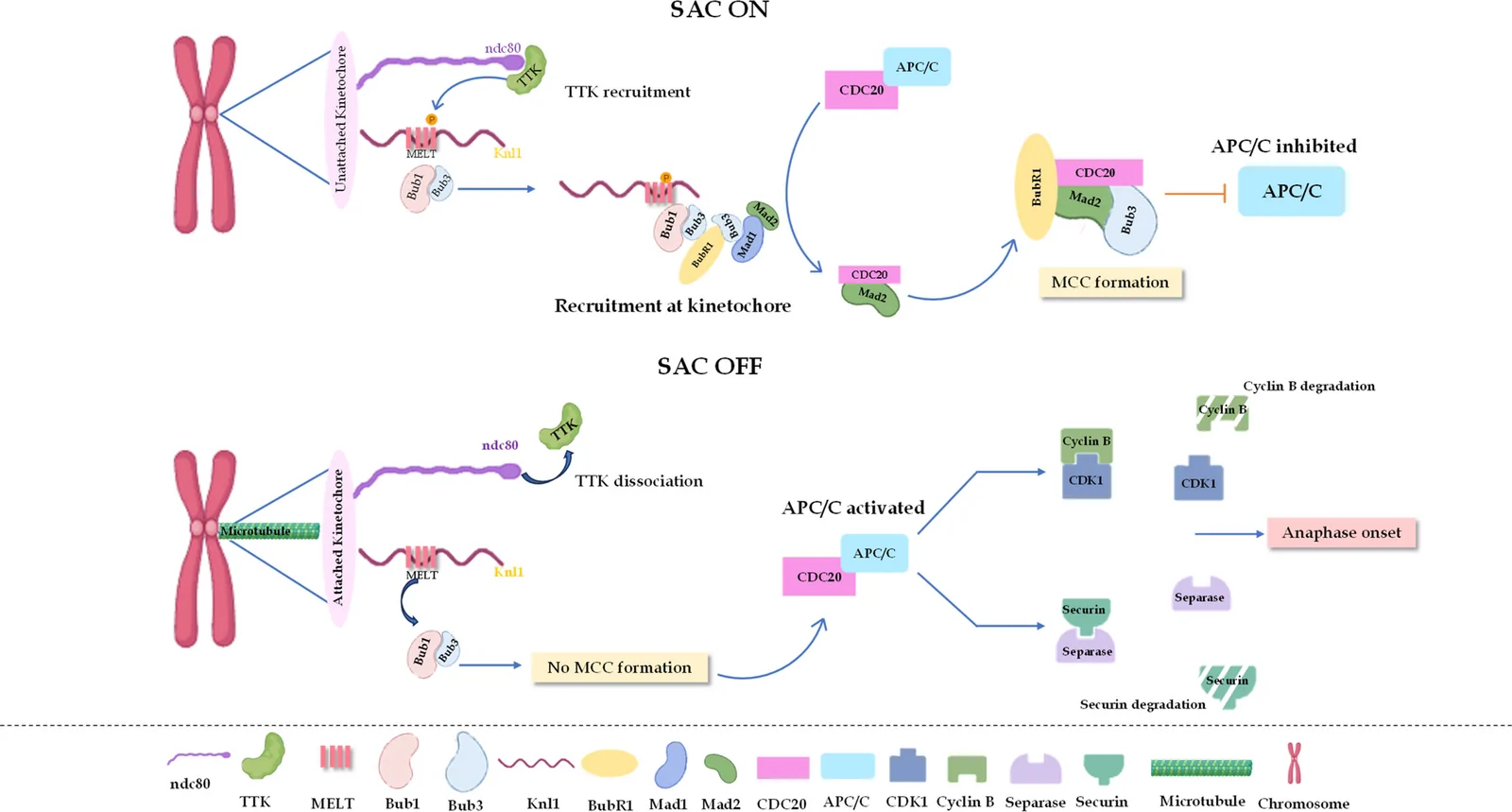
TTK-mediated regulation of spindle assembly checkpoint activation. Schematic representation of the role of TTK in coordinating spindle assembly checkpoint (SAC) signaling at kinetochores and ensuring accurate chromosome segregation. SAC ON: During early mitosis, when kinetochores are not properly attached to spindle microtubules, TTK is recruited to the kinetochore through interaction with the NDC80 complex. Activated TTK phosphorylates MELT motifs on KNL1, generating docking sites for checkpoint proteins including BUB3–BUB1 and BUB3–BUBR1 complexes. This scaffold facilitates recruitment of the MAD1–MAD2 complex, promoting conformational activation of MAD2 and subsequent assembly of the mitotic checkpoint complex (MCC), composed of BUBR1, BUB3, MAD2, and CDC20. The MCC binds to and inhibits the APC/C–CDC20 complex, preventing ubiquitination of securin and cyclin B, thereby blocking premature anaphase onset and ensuring proper chromosome alignment. SAC OFF: Upon successful kinetochore–microtubule attachment and generation of tension, TTK dissociates from kinetochores, leading to reduced phosphorylation of MELT motifs and disassembly of the checkpoint signaling platform. Consequently, recruitment of BUB and MAD proteins is reduced, leading to MCC disassembly. Free CDC20 then activates the APC/C, triggering ubiquitin-mediated degradation of securin and cyclin B. This leads to activation of separase and inactivation of CDK1, collectively driving the onset of anaphase and enabling faithful chromosome segregation

Once positioned at the kinetochore, TTK phosphorylates the MELT motifs within the scaffold protein KNL1 [42], generating high-affinity phospho-dependent docking sites for the BUB3–BUB1 complex [15]. This phosphorylation event establishes a hierarchical signaling platform that enables sequential recruitment of additional SAC components. Importantly, beyond MELT phosphorylation, TTK also phosphorylates key regulatory residues within BUB1’s central region, notably Ser 461 in human BUB1, which is required to stabilize the BUB1-MAD1 interaction and thereby promote efficient MAD1-MAD2 recruitment to unattached kinetochores [43, 44]. Once targeted, TTK further phosphorylates MAD1 at Thr716 in its C-terminal region to promote conformational activation of MAD2, a crucial step for the efficient O-MAD2-to-C-MAD2 conversion and robust mitotic checkpoint complex (MCC) assembly [14, 43, 45]. The MCC, composed of MAD2, BUBR1 (BUB1B), BUB3, and CDC20, acts as the diffusible inhibitory signal of the SAC. By binding to and sequestering CDC20, the MCC suppresses activation of the anaphase-promoting complex/cyclosome (APC/C), thereby preventing ubiquitination and degradation of Cyclin B and Securin [46]. Sustained Cyclin B levels maintain CDK1 activity, while intact Securin restrains Separase, collectively enforcing metaphase arrest until all chromosomes achieve proper bi-orientation. As microtubule attachments mature into stable end-on configurations, TTK dissociates from kinetochores, leading to reduced phosphorylation of KNL1 and to progressive disassembly of BUB and MAD complexes [39]. This attenuation of TTK activity terminates MCC production [47], allowing CDC20 to activate APC/C. Subsequent degradation of Cyclin B and Securin permits Separase activation, cohesin cleavage, and anaphase onset [48]. For instance, experimental inhibition of TTK with small-molecule inhibitors such as OSU-13 confirms its essential role in checkpoint maintenance, as pharmacological suppression disrupts MCC assembly and forces premature mitotic exit despite the presence of attachment defects [49].Taken together, as illustrated in Fig. 2 and supported by extensive mechanistic and functional studies, TTK operates as the central catalytic switch of the SAC-initiating checkpoint signaling at unattached kinetochores, amplifying inhibitory complex formation, integrating attachment error correction, and coordinating timely checkpoint silencing to ensure high-fidelity chromosome segregation and preservation of genomic stability.

### Role of TTK in Kinetochore-microtubule error correction

**Fig. 3:**
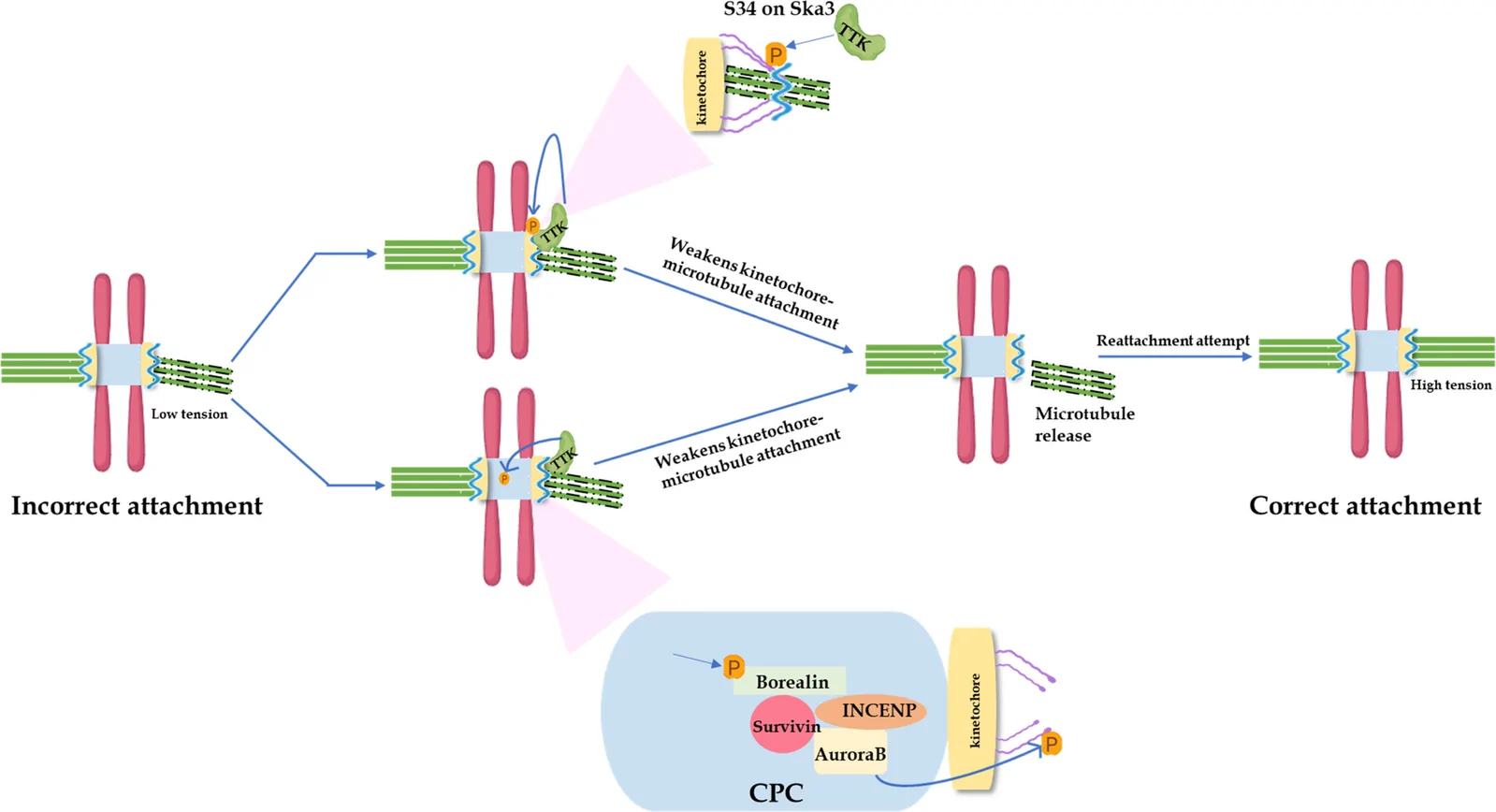
TTK-mediated kinetochore–microtubule error correction during mitosis. Figure illustrating the role of TTK in correcting improper kinetochore–microtubule attachments to ensure high-fidelity chromosome segregation during mitosis. Under low-tension conditions arising from erroneous attachments such as syntelic or merotelic orientations, TTK remains active at kinetochores and promotes error correction through two complementary mechanisms. First, TTK directly phosphorylates components of the Ska complex, particularly Ska3, which weakens the interaction between kinetochores and microtubules. This destabilization promotes microtubule detachment, enabling repeated cycles of attachment and reattachment until correct bipolar orientation is achieved. Second, TTK regulates the activity of the CPC by targeting its component Borealin, thereby facilitating proper localization and activation of Aurora B kinase at inner centromeres. Activated Aurora B phosphorylates multiple kinetochore substrates, further destabilizing incorrect microtubule attachments and enhancing error correction efficiency. Through this pathway, TTK indirectly amplifies Aurora B-mediated tension-sensing mechanisms that discriminate between correct and incorrect attachments

In addition to SAC signaling, extensive evidence indicates that TTK also plays a direct, mechanistically distinct role in correcting erroneous kinetochore–microtubule attachments [50]. TTK plays a central and multifaceted role in kinetochore–microtubule error correction by coordinating attachment sensing, microtubule-binding modulation, and chromosomal passenger complex (CPC) signaling to ensure accurate chromosome bi-orientation prior to anaphase onset**. **Figure 3 illustrates how TTK operates as a dynamic surveillance kinase that distinguishes improper attachments from correct end-on bipolar interactions and remodels kinetochore architecture accordingly. During early mitosis, chromosomes frequently establish lateral, monotelic or syntelic attachments that lack proper tension across sister kinetochores [51]. Under these low-tension conditions, TTK remains enriched at the outer kinetochore, where its kinase activity destabilizes erroneous kinetochore–microtubule interactions, allowing corrective reattachment attempts. By reducing attachment stability under improper geometric configurations, TTK promotes turnover of faulty microtubule interactions, thereby facilitating repeated cycles of attachment and detachment until correct amphitelic orientation is achieved [52, 53]. This dynamic instability is essential for maintaining attachment plasticity during prometaphase.

Furthermore, TTK also tightly controls the recruitment and function of the Ska complex, a key microtubule-stabilizing factor. The Ska complex enhances processive end-on microtubule binding and is critical for stabilizing mature kinetochore–microtubule attachments. TTK restricts premature Ska accumulation at kinetochores during early attachment stages, thereby preventing stabilization of erroneous interactions [52, 54]. Only when appropriate bipolar tension is generated, and microtubule attachment matures, does TTK dissociate from kinetochores, allowing Ska recruitment and consolidation of stable end-on attachments [52]. Thus, TTK establishes a temporal hierarchy in which destabilization precedes stabilization, ensuring that only correctly orientated attachments become reinforced.

Beyond outer kinetochore modulation, TTK also influences inner centromere signaling by regulating the CPC. TTK phosphorylates Borealin, a core CPC component, thereby modulating Aurora B kinase activity and promoting efficient correction of misattachments [55]. Aurora B functions as a tension-sensitive kinase that destabilizes low-tension attachments by regulating CPC activity. TTK strengthens the biochemical gradient that distinguishes properly from improper microtubule attachments. This coordination between TTK and Aurora B integrates mechanical tension sensing with phosphorylation-based signaling to enhance correction efficiency. Importantly, the association of TTK with kinetochores is highly sensitive to attachment maturation. Stable end-on microtubule occupancy reduces TTK recruitment, leading to diminished phosphorylation of outer kinetochore substrates and attenuation of destabilizing signals [39]. This displacement marks a critical transition from an error-correction–dominant state to a stabilization phase, ultimately enabling SAC silencing and progression into anaphase once all chromosomes are properly bi-oriented.

Together, TTK functions as a central coordinator of kinetochore error correction. It destabilizes incorrect attachments by modulating Ndc80, temporally regulates Ska-mediated stabilization, enhances Aurora B-dependent correction via Borealin phosphorylation, and dynamically responds to tension-dependent attachment maturation. By integrating structural, mechanical, and kinase-driven signaling pathways, TTK ensures that only correctly attached and tension-bearing chromosomes proceed to segregation, thereby safeguarding chromosomal stability and preventing aneuploidy.

## Non-canonical roles of TTK: from genome surveillance to tumor adaptation

Although TTK has been implicated in multiple oncogenic processes, these functions are not equivalent in their contribution to tumor biology. Its canonical role in SAC regulation represents the primary molecular function responsible for maintaining chromosome segregation fidelity [4]. Dysregulation of this checkpoint promotes CIN, which serves as the central initiating event driving genomic stress. Persistent CIN subsequently activates non-canonical adaptive pathways, including DNA damage response (DDR), epithelial-mesenchymal transition (EMT), apoptosis regulation, immune modulation, and therapy resistance. Thus, rather than representing a single hierarchical cascade in which all non-canonical functions are downstream consequences of SAC dysfunction, these processes reflect an interconnected network in which TTK can act both upstream and downstream of cellular stress. These non-canonical activities shape cell fate decisions and tumor behavior, particularly under conditions of genotoxic, oxidative, and oncogenic stress.

### TTK as a modulator of DDR signaling

**Fig. 4:**
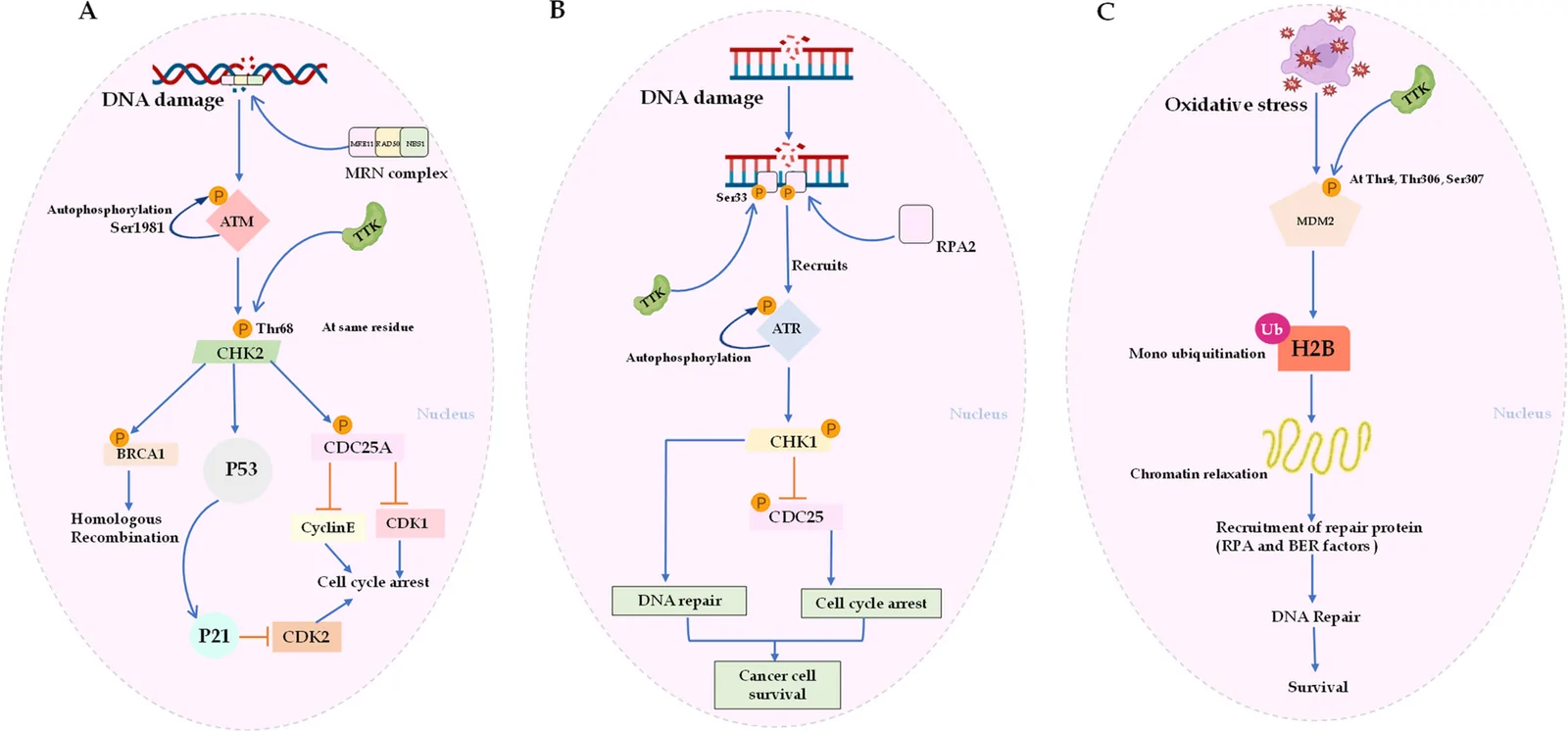
TTK-mediated regulation of DNA damage response pathways. **A** TTK in ATM-CHK2 signaling during double-strand break response: Upon induction of DNA DSBs, the MRN complex (MRE11–RAD50–NBS1) facilitates ATM activation via autophosphorylation at Ser1981. Activated ATM phosphorylates CHK2 at Thr68, initiating checkpoint signaling. TTK is shown to phosphorylate CHK2 at the same residue, suggesting a supportive role in checkpoint amplification. Activated CHK2 promotes downstream signaling to p53, leading to p21 transcriptional activation. p21 inhibits CDK2 activity, while CHK2-mediated regulation of CDC25A further suppresses CDK1/Cyclin E activity, collectively resulting in cell-cycle arrest and allowing time for homologous recombination repair via BRCA1. **B** TTK regulation of replication stress response via RPA2–ATR–CHK1 axis: Replication stress leads to accumulation of single-stranded DNA (ssDNA), which is coated by RPA. TTK phosphorylates RPA2 (Ser33), facilitating efficient recruitment and activation of ATR. ATR undergoes autophosphorylation and activates CHK1 by phosphorylating it. Activated CHK1 inhibits CDC25 phosphatases, preventing activation of cyclin-dependent kinases and enforcing S-phase or G2/M checkpoint arrest. This pathway promotes DNA repair and supports cancer cell survival under conditions of replication stress. **C** TTK-mediated chromatin modulation under oxidative stress: Under oxidative stress conditions, TTK phosphorylates MDM2 at Thr4, Thr306, and Ser307. This modification is associated with enhanced monoubiquitination of histone H2B, leading to chromatin relaxation. The relaxed chromatin state facilitates recruitment of DNA repair proteins, including RPA and base excision repair (BER) factors, thereby promoting efficient DNA repair and cellular survival

One of the earliest indications that TTK functions beyond mitosis emerged from studies of the DDR. As shown in Fig. 4A, in response to DSBs, checkpoint signaling is initiated by the MRN (MRE11-RAD50-NBS1) complex, which recruits and activates ATM through autophosphorylation at Ser1981 [56]. Activated ATM phosphorylates multiple substrates, including checkpoint kinase 2 (CHK2) at Thr68, thereby triggering downstream cell-cycle arrest pathways [57]. Importantly, TTK directly participates in this signaling cascade rather than acting solely downstream of ATM. Biochemical and cellular analyses demonstrate that TTK physically associates with CHK2 and directly phosphorylates it at Thr68 following genotoxic stress, providing direct mechanistic evidence for its role in DDR signaling. Loss of TTK markedly reduces CHK2 Thr68 phosphorylation, resulting in incomplete checkpoint activation [58]. These findings establish TTK as a cooperative amplifier of ATM signaling, ensuring robust CHK2 activation rather than substituting for ATM function. Once activated, CHK2 activates and phosphorylates p53 and CDC25A, leading to induction of p21, inhibition of CDK1/CDK2 activity, and enforcement of G2/M arrest [59]. Through this mechanism, TTK strengthens DNA damage checkpoint fidelity and promotes genome stabilization by prolonging cell-cycle arrest and allowing time for repair [58].

An additional layer of TTK involvement in the DNA damage response is illustrated in Fig. 4B, which depicts its role in RPA–mediated replication stress signaling. RPA is a heterotrimeric single-stranded DNA–binding complex that stabilizes exposed DNA during replication stress and serves as a platform for the recruitment of DNA repair and checkpoint proteins. RPA2 undergoes phosphorylation in response to replication stress, a modification required for efficient activation of downstream ATR-CHK1 checkpoint signaling [59–61].

Genetic depletion and pharmacological inhibition studies indicate that TTK promotes RPA2 phosphorylation, although the precise molecular mechanism remains under investigation [62]. Loss of TTK activity reduces RPA2 phosphorylation and compromises ATR-dependent checkpoint signaling, ultimately impairing stabilization of replication forks and increasing genomic instability [62]. Through this mechanism, TTK links spindle checkpoint signaling with replication stress responses, reinforcing genome stability under conditions of DNA damage. The mechanistic framework of the TTK-RPA2-ATR signaling axis has been further summarized in recent studies examining replication stress associated checkpoint regulation [63].

TTK also contributes to DDR through a mechanistically distinct pathway centered on chromatin remodeling during oxidative stress. Under conditions of oxidative DNA damage, TTK phosphorylates the E3 ubiquitin ligase MDM2 at Thr4, Thr306, and Ser307 [64]. These phosphorylation events stabilize MDM2 and enhance its ligase activity toward histone H2B, resulting in mono-ubiquitination at Lys120 [65]. This modification induces localized chromatin relaxation, increasing accessibility of damaged DNA regions to repair machinery. As a consequence, replication protein A (RPA) and base excision repair (BER) factors are more efficiently recruited, facilitating repair of oxidative lesions [66], as illustrated in Fig. 4C. Notably, this MDM2 function is independent of p53 degradation, highlighting a chromatin-centered role distinct from its classical function [67]. Loss of TTK disrupts this repair axis, leading to defective chromatin remodeling, impaired recruitment of repair factors, and heightened sensitivity to oxidative stress [64].

Hence, these findings position TTK as a non-canonical regulator of DDR that operates at multiple levels, coordinating checkpoint signaling and chromatin accessibility to preserve genome integrity under diverse forms of genotoxic stress.

### Coupling genome stress to cellular plasticity: TTK and EMT

Beyond genome maintenance, TTK has emerged as a key regulator of EMT, linking stress-adaptive signaling to tumor progression. EMT is characterized by loss of epithelial identity, acquisition of mesenchymal features, increased motility, and enhanced metastatic potential. Increasing evidence indicates that TTK regulates EMT-associated markers and invasive behavior, supporting an indirect role in EMT rather than direct phosphorylation of EMT transcription factors [68]. In several cancer models, elevated TTK expression enhances activation of the PKCα-ERK1/2-MAPK axis. TTK upregulates PKCα expression, which in turn promotes ERK1/2 phosphorylation and downstream transcriptional programs associated with proliferation, dedifferentiation, and cellular plasticity. Although ERK is not a direct substrate of TTK, PKCα-dependent amplification of MAPK signaling provides a mechanism by which TTK indirectly drives EMT-associated transcriptional reprogramming [69].

**Fig. 5:**
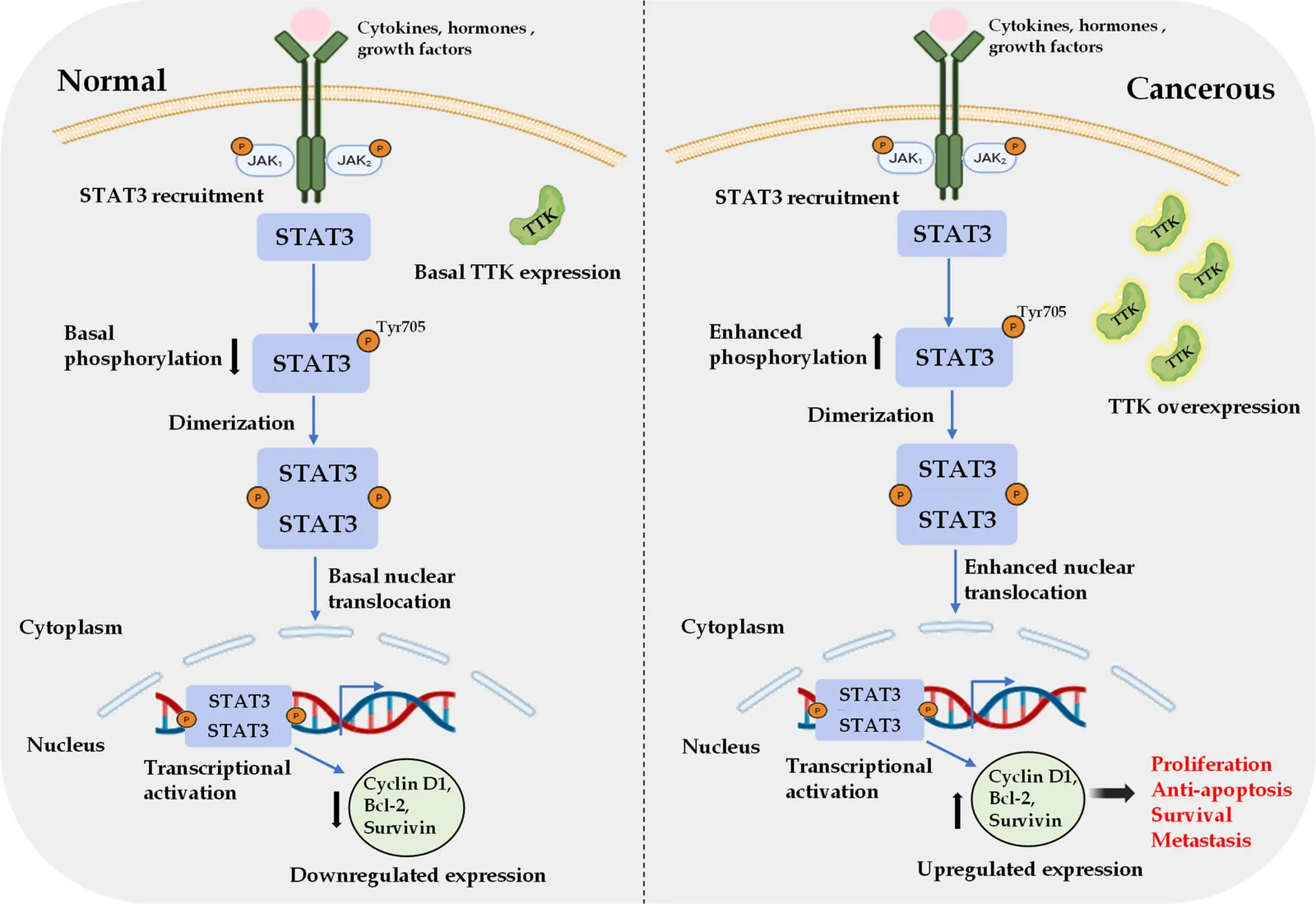
TTK-associated modulation of the JAK/STAT3 signaling axis. Normal physiological condition: In normal cells, extracellular signals, such as cytokines, hormones, or growth factors, activate receptor-associated kinases JAK1 and JAK2, leading to the phosphorylation of STAT3 at Tyr705. Under basal conditions, TTK expression remains low. STAT3 activation is therefore limited, resulting in controlled dimerization and modest nuclear translocation. In the nucleus, STAT3 drives basal transcription of target genes, including Cyclin D1, BCL-2, and Survivin, thereby maintaining regulated cell proliferation and survival. This ensures balanced cellular homeostasis without aberrant signaling. Cancerous condition: In cancer cells, TTK is overexpressed, as indicated in the schematic. This is associated with enhanced STAT3 phosphorylation at Tyr705 and increased STAT3 activation. Phosphorylated STAT3 dimerizes and undergoes increased nuclear translocation, leading to elevated transcriptional activity. Consequently, expression of STAT3 target genes, including *Cyclin D1*, *BCL-2*, and *Survivin*, is upregulated. This transcriptional shift supports key oncogenic processes, including increased proliferation, resistance to apoptosis, enhanced survival, and metastatic potential

TTK has also been linked to activation of the STAT3 signaling pathway, providing an additional mechanism through which it contributes to tumor progression and cellular plasticity, as depicted in Fig. 5. Under normal conditions, STAT3 signaling is maintained at a basal level and is initiated when cytokines or growth factors, such as IL-6, bind their receptors, leading to activation of receptor-associated JAK kinases. JAK-mediated phosphorylation of STAT3 promotes its dimerization and nuclear translocation, where it regulates transcription of genes involved in cell survival and proliferation, including Cyclin D1, Bcl-2, and Survivin [70]. Functional studies using TTK overexpression and knockdown models demonstrated increased STAT3 phosphorylation following TTK activation. However, direct phosphorylation of STAT3 by TTK has not been demonstrated, suggesting an indirect regulatory mechanism [71]. Through sustained STAT3 activation, TTK drives increased transcription of target genes, including Cyclin D1, Bcl-2, and Survivin, which promote proliferation, survival, and tumor progression.

Consistent with these findings, persistent STAT3 signaling is known to regulate EMT-associated transcriptional networks that support tumor invasion, immune modulation, and metastatic dissemination [68]. Thus, TTK-driven amplification of STAT3 signaling provides a potential mechanism linking mitotic checkpoint dysregulation with transcriptional pathways that promote mesenchymal plasticity and cancer progression. Furthermore, evidence from pharmacological inhibition and genetic silencing studies suggests that TTK positively regulates the PI3K-AKT-mTOR pathway. However, whether AKT is a direct substrate of TTK remains unresolved. In cancer models including gastric, Liver cancer TTK overexpression enhances AKT phosphorylation, while its depletion attenuates AKT activity [72, 73]. Activated AKT stimulates mTOR signaling, leading to phosphorylation of S6K and inhibition of 4EBP1, thereby promoting protein synthesis and translational stabilization of EMT-associated transcription factors such as SNAI1. Through this mechanism, TTK supports long-term maintenance of mesenchymal identity and invasive behavior.

### TTK Regulation of c-Abl–Dependent apoptotic signaling under oxidative stress

c-Abl is a stress-responsive non-receptor tyrosine kinase that becomes activated following exposure to DNA-damaging agents such as ionizing radiation and chemotherapeutic drugs [74]. Beyond its activation, c-Abl function is critically regulated by subcellular localization, which determines its ability to induce apoptotic signaling [75]. Under basal conditions, TTK directly phosphorylates c-Abl at Thr735, promoting its interaction with 14–3-3 proteins and enforcing cytoplasmic retention [76]. This phosphorylation-dependent sequestration represents a key regulatory checkpoint that restrains c-Abl activity and prevents unintended activation of stress-induced apoptotic pathways [16]. By maintaining c-Abl in the cytoplasm, TTK effectively suppresses nuclear apoptotic signaling and contributes to cell survival under non-stress conditions [76].

Upon oxidative stress or DNA damage, activation of the JNK pathway serves as a critical switch controlling c-Abl localization [77]. JNK phosphorylates 14–3-3 proteins, leading to dissociation of the c-Abl–14–3-3 complex and release of c-Abl from cytoplasmic retention [77, 78]. This release permits rapid relocalization of c-Abl, highlighting a tightly controlled mechanism through which stress signals are translated into changes in protein localization [75]. Following release, c-Abl translocates to the nucleus, where it promotes apoptotic signaling through phosphorylation and activation of the transcription factor p73 [79]. Activated p73 then drives the transcriptional upregulation of pro-apoptotic genes, including BAX, BAK, and PUMA, thereby facilitating apoptotic cell death [80]. In the nucleus, c-Abl also promotes apoptosis in part by stabilizing pro-apoptotic transcription factors such as Pitx1 [81]. Additionally, c-Abl has been implicated in amplifying apoptotic signaling cascades by integrating DNA damage and oxidative stress responses, thereby reinforcing cell fate decisions under severe stress conditions [75].

**Fig. 6:**
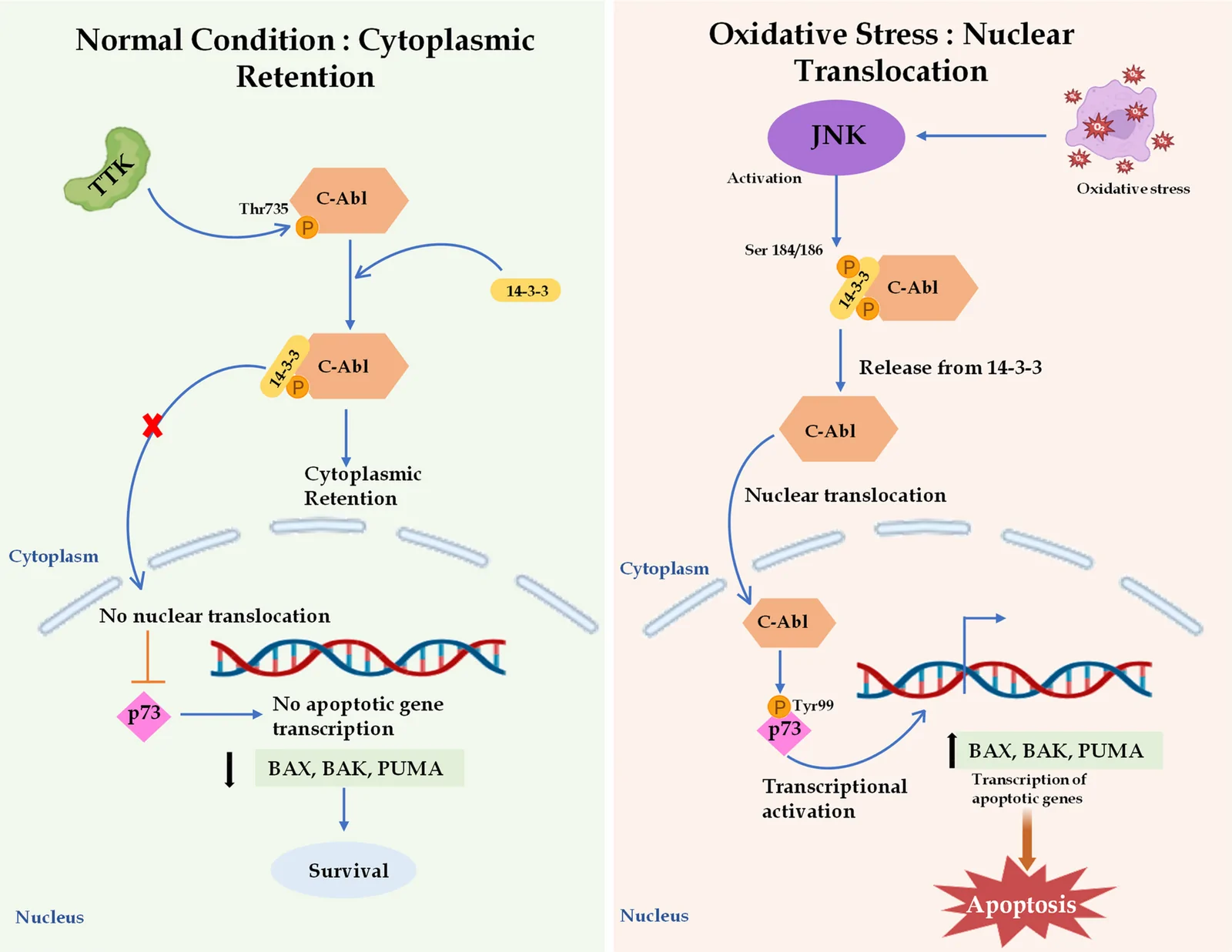
TTK-associated regulation of c-Abl subcellular localization and stress-induced apoptotic signaling. Normal physiological condition (cytoplasmic retention): Under basal conditions, TTK is shown promoting phosphorylation of c-Abl at Thr735, which facilitates its interaction with 14–3-3 adaptor proteins. This interaction results in cytoplasmic retention of c-Abl and prevents its nuclear translocation. Consequently, c-Abl–dependent activation of p73 is not induced, and transcription of pro-apoptotic genes remains low, as indicated by reduced expression of BAX, BAK, and PUMA. This state is associated with maintenance of cell survival. Oxidative stress condition (nuclear translocation): In response to oxidative stress, JNK is activated and phosphorylates c-Abl (Ser184/186 as indicated), promoting dissociation from 14–3-3 proteins. Released c-Abl translocates to the nucleus, where it phosphorylates and activates p73 (Tyr99). Activated p73 drives transcriptional upregulation of pro-apoptotic genes, including BAX, BAK, and PUMA. This transcriptional activation leads to induction of apoptosis

In addition to its regulation of nuclear apoptotic signaling, emerging evidence indicates that TTK also modulates mitochondrial apoptosis through stress-adaptive mechanisms. Pharmacological inhibition of TTK has been shown to induce caspase-dependent apoptosis and mitochondrial dysfunction, supporting a pro-survival role of TTK in cancer cells [82]. Complementing this, recent findings employing genetic depletion and pharmacological inhibition of TTK demonstrate that TTK promotes mitophagy through ULK1 phosphorylation, although additional validation across different tumor models is still required [83]. This mitophagy-mediated mitochondrial quality control prevents cytochrome c release and suppresses activation of the intrinsic apoptotic pathway [84]. Loss of TTK disrupts this protective mechanism, leading to mitochondrial stress and increased apoptotic susceptibility [83]. Together, these observations suggest that TTK suppresses apoptosis through a dual regulatory framework that involves both nuclear signaling and mitochondrial homeostasis, thereby enabling tumor cell survival under oxidative and metabolic stress. These findings demonstrate that TTK suppresses apoptosis by coordinating the regulation of nuclear and mitochondrial pathways. While TTK-mediated phosphorylation maintains c-Abl in an inactive cytoplasmic state to restrain nuclear apoptotic signaling (Fig. 6) [77, 78]. Emerging evidence indicates that TTK also preserves mitochondrial integrity through mitophagy-dependent mechanisms, thereby limiting intrinsic apoptotic activation under stress conditions.

### Context-dependent modulation of p53 signaling by TTK

TTK has also been implicated in regulating p53 signaling, linking mitotic checkpoint control with genome surveillance pathways [85]. Under physiological conditions, TTK directly phosphorylates p53 at Thr18, a modification that disrupts its interaction with the E3 ubiquitin ligase MDM2 and prevents p53 degradation [86]. This phosphorylation event stabilizes p53 and enhances its transcriptional activity, leading to the induction of downstream targets such as p21 and LATS2, which mediate postmitotic checkpoint arrest [86, 87]. Through this mechanism, TTK links SAC signaling to p53-dependent genome surveillance, contributing to genomic stability by coordinating DNA damage responses and preventing the propagation of cells with mitotic defects [64].

In contrast, the outcome of TTK inhibition is itself dependent on TP53 mutation status: in TP53-wild-type, chromosomally unstable tumor cells, TTK inhibition triggers p53-mediated post-mitotic cell death, whereas TP53-mutant tumor cells tolerate the resulting aneuploidy and can survive or acquire drug resistance [88]. Pharmacological inhibition of TTK results in activation of p53 signaling and induction of apoptosis, indicating that elevated TTK activity restrains effective p53-dependent cell death [89]. These findings suggest that TTK regulates p53 signaling in a context-dependent manner, functioning as a genome surveillance kinase under normal conditions while contributing to cancer cell survival when aberrantly upregulated. Together, these findings establish TTK as a multifunctional regulator that integrates genome surveillance, stress response, and cellular plasticity beyond its canonical mitotic role. By coordinating DDR, apoptotic signaling, and EMT-associated pathways, TTK enables adaptive responses to oncogenic stress that are increasingly co-opted during tumor progression. This expanded functional scope provides a framework for understanding the diverse roles of TTK across cancer types.

## TTK in various cancers

TTK has emerged as a cancer-relevant kinase whose functions extend far beyond its canonical role in mitotic surveillance. Pan-cancer transcriptomic and functional studies consistently demonstrate that TTK expression is elevated in malignant tissues compared with normal counterparts and is associated with aggressive tumor behavior and adverse clinical outcomes [90, 91], as depicted in Table 1. Importantly, elevated TTK expression alone should not be considered synonymous with reliance on functional TTK, as expression may also reflect proliferative activity or cell-cycle state. However, functional studies across multiple tumor models demonstrate that TTK suppression compromises cancer cell viability and proliferative capacity, whereas normal cells exhibit greater tolerance to partial checkpoint disruption [115]. These observations support a model in which tumor cells develop a selective dependency on TTK activity to buffer the deleterious consequences of genomic instability. In this framework, TTK functions as a stress-adaptive node that integrates mitotic control with non-mitotic signaling pathways, providing a unifying explanation for its broad relevance across cancer types.

**Table 1:** Pan-cancer expression profile and prognostic significance of TTK across human malignancies

S No	Cancer Type	TTK Expression Level (vs. normal)	Survival Trend (High TTK vs. Low TTK)	Status Specific Survival Endpoints Addressed	Evidence Level	References
	Breast Cancer (TNBC)	Overexpressed	Poorer prognosis; increased invasion & EMT	Overall Survival (OS); therapy resistance	Prognostic (patient cohort)	[92] [93]
	Triple-Positive Breast Cancer	Elevated	Poor prognosis	OS	Prognostic (patient cohort)	[94]
	HER2 + Breast Cancer	Elevated	Poor prognosis; therapy resistance	OS; response to targeted therapy	Prognostic (patient cohort)	[95]
	NSCLC	Overexpressed	Poor prognosis; aggressive behaviour	OS; progression correlation	Prognostic (patient cohort)	[96]
	KRAS–LKB1 Mutant Lung Cancer	Functionally critical	Sensitization to immunotherapy upon inhibition	Immunotherapy response	Functional (genetic/pharmacological)	[97]
	Glioma/Glioblastoma	Elevated	Reduced survival; chemoresistance	OS; temozolomide response	Prognostic (patient cohort)	[98] [99] [100]
	Gastric Cancer	Overexpressed	Poor prognosis; aggressive growth	OS; apoptosis/proliferation markers	Prognostic (patient cohort)	[101]
	Colorectal Cancer	Elevated	Poor prognosis	OS; tumor differentiation status	Prognostic (patient cohort)	[102]
	Hepatocellular Carcinoma	Upregulated	Poor prognosis; enhanced migration	OS; tumor progression	Prognostic + Functional	[103] [104]
	Bladder Cancer	Overexpressed	Aggressive phenotype; metastatic behavior	OS; mitotic dependency	Functional (genetic/pharmacological)	[105] [83]
	Ovarian Cancer	Elevated	Poor prognosis; cisplatin & olaparib resistance	OS; chemotherapy response	Prognostic (patient cohort)	[106]
	Pancreatic Cancer	Overexpressed	Poor prognosis; mitotic dependency	OS; proliferation index	Functional (genetic/pharmacological)	[107, 108]
	Prostate Cancer	Elevated	Tumor progression	OS; proliferation markers	Prognostic (patient cohort)	[109]
	Endometrial Cancer	Elevated	Poor prognosis; immune infiltration	OS; immune association	Prognostic (patient cohort)	[10]
	Liver Cancer (Metastatic Spread)	Elevated	Associated with tumor dissemination	OS; intrahepatic spread	Prognostic (patient cohort)	[110]
	Esophageal Cancer	Overexpressed	Poor prognosis; oncogenic signaling	OS	Prognostic (patient cohort)	[111]
	Cervical Cancer	Increased	Associated with adverse clinicopathological features	OS; clinicopathological correlation	Prognostic (patient cohort)	
	Papillary Thyroid Cancer	Elevated	Tumor dedifferentiation; immune infiltration	OS; risk stratification	Prognostic (patient cohort)	[112]
	Osteosarcoma	Increased	Disease progression	OS	Prognostic (patient cohort)	[113]
	Oral Squamous Cell Carcinoma	Elevated	High-risk subtype; poor prognosis	OS; mTORC1/NF-κB activation	Prognostic (patient cohort)	[114]

### Breast cancer

In triple-negative breast cancer (TNBC), elevated TTK expression promotes mesenchymal signaling and invasive behavior, linking its mitotic functions to EMT-associated programs that drive tumor aggressiveness [68]. Mechanistic studies further demonstrate that TTK regulates cell-cycle progression and mitotic fidelity in TNBC cells, conferring survival advantages under conditions of mitotic stress and uncontrolled proliferation [92]. Beyond TNBC, TTK expression also carries prognostic significance in other breast cancer subtypes. In triple-positive breast cancer, elevated TTK levels correlate with poor prognosis and increased proliferative and invasive capacity [94]. In HER2-positive breast cancer, TTK has been implicated in promoting cell migration, resistance to targeted therapies, and altered apoptotic responses by modulating the AKT–mTOR signaling axis, highlighting non-canonical signaling roles beyond checkpoint control [95]. The subtype-specific modes of TTK exploitation underscore its potential as a therapeutic vulnerability in aggressive and treatment-refractory disease.

### Lung cancer

In lung cancer, extensive evidence supports a central role for TTK in tumor progression, therapeutic vulnerability, and immune modulation. Pharmacological inhibition of TTK induces severe mitotic defects, polyploidy, and apoptosis in lung cancer models, demonstrating a strong reliance of these tumors on intact TTK-mediated mitotic surveillance [116]. Disruption of this checkpoint dependency overwhelms lung cancer cells' tolerance to CIN, leading to cell death rather than sustained proliferation. In non-small cell lung cancer (NSCLC), elevated TTK expression consistently correlates with poor prognosis and aggressive clinical behavior. Integrative bioinformatics analyses combined with experimental validation support its utility as a prognostic biomarker [96]. Mechanistically, TTK upregulation has been linked to enhanced oncogenic signaling, including neurotensin- and DPYSL3-driven pathways that elevate TTK expression and promote tumor growth [117]. Beyond tumor cell–intrinsic effects, recent studies reveal an unexpected role for TTK in shaping tumor immunogenicity. In KRAS-LKB1 mutant lung cancers, MPS1 inhibition enhances antitumor immune responses by increasing tumor immunogenicity, thereby sensitizing tumors to immune-mediated clearance [97]. This finding expands the functional relevance of TTK in lung cancer beyond mitotic control and highlights its potential integration with immunotherapeutic strategies.

### Glioma

Glioma, particularly glioblastoma, is characterized by extreme genomic instability and therapeutic resistance, and recent studies identify TTK as a key contributor to this aggressive phenotype. TTK promotes temozolomide resistance by inducing protective autophagy, enabling glioblastoma cells to survive chemotherapeutic stress. Inhibition of TTK disrupts this adaptive response and restores drug sensitivity, indicating that TTK supports tumor survival through stress-response pathways in addition to its role in mitotic regulation [98]. Clinical and transcriptomic analyses further underscore the pathological relevance of TTK in glioma. Elevated TTK expression correlates with earlier symptom onset, increased tumor aggressiveness, and reduced patient survival [97, 117]. Incorporation of mitotic checkpoint gene signatures, including TTK, improves prognostic stratification in glioma, reinforcing the link between checkpoint dysregulation and disease outcome [118]. Pharmacological inhibition with selective MPS1 inhibitors, such as MPS1-IN-3, induces catastrophic chromosome segregation defects and sensitizes glioblastoma cells to antimitotic agents, highlighting a selective reliance on TTK-mediated mitotic surveillance [119].

### Gastric cancer

In gastric cancer, TTK contributes to tumor progression through both mitotic and signaling-based mechanisms. Elevated TTK expression promotes cell proliferation and suppresses apoptosis through activation of the AKT-mTOR signaling pathway, linking checkpoint control to pro-survival signaling networks [72]. This signaling role provides a mechanistic basis for the aggressive growth behavior observed in TTK-high gastric tumors. Pharmacological targeting further supports the functional importance of TTK in gastric cancer. Reversine and selective TTK inhibitors suppress proliferation, invasion, and migration while inducing apoptosis, demonstrating a dependency on TTK activity for both growth and metastatic traits [120, 121]. In addition, genetic association studies link polymorphisms in mitotic segregation genes, including TTK, to increased gastric cancer risk, implicating checkpoint dysregulation in tumor susceptibility [101].

### Colorectal cancer

In colorectal cancer (CRC), TTK functions as a key integrator of cell-cycle regulation and oncogenic signaling. Elevated TTK expression predicts poor prognosis and is associated with enhanced proliferation and altered differentiation [69]. Mechanistically, TTK activates multiple growth-promoting pathways, including the PKCα-ERK1/2 and PI3K-AKT signaling pathways, reinforcing its role beyond a classical checkpoint kinase [69]. TTK also operates within broader spindle-associated regulatory networks in CRC. Targeting the TPX2-TTK axis disrupts mitotic progression and suppresses tumorigenic capacity, indicating that CRC cells rely on coordinated regulation of spindle assembly and checkpoint signaling for sustained growth [102]. These findings position TTK as both a prognostic marker and a rational therapeutic target in colorectal cancer.

### Hepatocellular carcinoma

In hepatocellular carcinoma (HCC), TTK supports tumor progression through a dual mechanism involving mitotic integrity and oncogenic signaling. TTK is markedly upregulated in HCC tissues and contributes directly to tumor cell growth and survival [103]. Suppression of TTK disrupts mitotic progression, induces chromosome segregation defects, and reduces proliferative capacity, indicating a strong dependency on intact TTK function [104]. Beyond mitosis, TTK promotes HCC cell proliferation and migration through activation of the AKT pathway, linking checkpoint control to invasive behavior [73]. This dual functionality explains how elevated TTK expression drives both uncontrolled cell division and enhanced tumor aggressiveness, reinforcing its relevance as a targetable vulnerability in liver cancer.

### Bladder cancer

In bladder cancer, TTK functions as a critical regulator of proliferation, invasion, and stress adaptation. TTK promotes tumor cell proliferation and migration and drives EMT, linking mitotic checkpoint dysregulation to enhanced metastatic behaviour [105]. Beyond its canonical SAC role, TTK supports mitochondrial survival pathways. It promotes mitophagy through ULK1 phosphorylation and regulates pre-mRNA splicing to suppress mitochondrial apoptosis, thereby enhancing tumor cell survival under stress conditions [83]. Post-transcriptional control further contributes to TTK dysregulation, as miR-582-5p directly targets TTK and inhibits bladder tumorigenesis [122]. Loss of this regulatory axis facilitates sustained TTK expression and tumor progression. Additionally, alterations, including mutations and loss of heterozygosity, in TTK and related checkpoint genes underscore the contribution of checkpoint pathway disruption to bladder cancer pathogenesis [123]. Therapeutically, bladder cancer cells exhibit dependence on TTK activity. The dual TTK/PLK1 inhibitor BAL0891 induces mitotic catastrophe and significantly suppresses tumor cell viability, highlighting TTK as a potential targetable vulnerability [124].

### Ovarian cancer

In ovarian cancer, TTK emerges as a critical regulator of tumor progression and therapeutic resistance. Elevated TTK expression promotes malignant behaviors, including proliferation, migration, and invasion, and is subject to post-transcriptional regulation by miR-582-5p [106]. Loss of this regulatory control contributes to sustained TTK expression and aggressive tumor phenotypes. A defining feature of TTK function in ovarian cancer is its role in chemoresistance. TTK is significantly upregulated in cisplatin-resistant ovarian tumors, and its inhibition restores cisplatin sensitivity, establishing TTK as a potential therapeutic target in drug-refractory disease [125]. Mechanistically, TTK inhibition enhances cisplatin responsiveness in high-grade serous ovarian carcinoma by modulating the mTOR/autophagy pathway, linking checkpoint control to stress-adaptive survival signaling [126].

Beyond platinum resistance, TTK also promotes resistance to PARP inhibition. TTK activates ATR via RPA2 phosphorylation, thereby enhancing DNA damage response signaling and promoting olaparib resistance [62] This finding positions TTK as a key integrator of mitotic regulation and replication stress responses in ovarian cancer. Therapeutically, multi-target strategies further support TTK vulnerability. In-silico analyses identify NSC777201 as a potential inhibitor targeting TTK along with NEK2 and CDK1, reinforcing the rationale for combinatorial targeting of mitotic kinases in ovarian carcinoma [127].

### Other cancers

Apart from the cancer types discussed above, TTK has also been implicated in a broad spectrum of additional malignancies, further underscoring its pan-cancer relevance. In pancreatic cancer, TTK plays a key role in sustaining proliferation and survival, and both genetic silencing and pharmacological inhibition impair tumor cell growth by inducing lethal CIN [107, 108], highlighting a strong dependency on intact SAC signaling. Similarly, in prostate cancer, suppression of TTK significantly inhibits tumor proliferation and progression [109], reinforcing its role as a driver of cell-cycle advancement. In endometrial cancer, elevated TTK expression is associated with poor prognosis and increased immune cell infiltration [10], and integrated multi-omics analyses identify TTK as a novel activator of EMT, linking checkpoint dysregulation to invasive behavior [128]. Prognostic modeling further recognizes TTK, together with CDC25A and ESPL1, as part of a high-risk biomarker signature [129]. In liver cancer, TTK contributes to intrahepatic tumor spread [110], while in esophageal cancer, it cooperates with Annexin A2 to accelerate tumor progression by activating the AKT/mTOR signaling pathway [111], demonstrating non-canonical signaling functions beyond mitosis.

Moreover, increased TTK expression correlates with adverse clinicopathological features in cervical cancer [130], tumor dedifferentiation, and immune infiltration in papillary thyroid cancer [112], and disease progression in osteosarcoma [113]. In oral squamous cell carcinoma, TTK defines a high-risk subtype characterized by dual activation of mTORC1 and NF-κB signaling pathways [114], further linking mitotic kinase dysregulation to oncogenic and inflammatory signaling networks. Collectively, these findings reinforce a unifying model in which TTK functions as a multifunctional regulator integrating chromosomal stability, oncogenic signaling, EMT programs, and immune interactions across diverse tumor types.

## Therapeutic landscape: targeting TTK

Pharmacological strategies targeting TTK can be broadly classified into direct inhibition of TTK kinase activity, indirect disruption of TTK-dependent signaling through associated SAC kinases, and combination approaches that exploit mitotic stress and DNA damage vulnerabilities (Table 2). The rationale for targeting TTK pharmacologically is based on the dose-dependent relationship between CIN and tumor cell fitness. Moderate levels of CIN are thought to promote tumor evolution by generating karyotypic diversity, intratumoral heterogeneity, and adaptive potential, thereby facilitating tumor progression, metastasis, and therapeutic resistance. However, this relationship is not linear. Once CIN exceeds a critical threshold, the cumulative burden of chromosome mis-segregation becomes incompatible with cell survival, leading to catastrophic genomic imbalance, mitotic catastrophe, and apoptotic cell death [131, 132]. Consistent with this concept, genetic studies using TTK dosage-controlled mouse models have demonstrated that the oncogenic consequences of CIN are themselves level-dependent, with moderate CIN promoting tumorigenesis, whereas excessive CIN suppresses tumor growth by exceeding the tolerable limit of genomic instability [133].

**Table 2:** Direct inhibitors of TTK: mechanisms and pharmacological characteristics

**S. No.**	**Inhibitor Name**	**Structure**	**Type of Cancer**	**Clinical Trial**	**Evidence Level**
1	**CFI-402257**		HER2-Negative Breast Cancer	Phase 1 Phase 2 NCT03568422	In vivo validation, Clinical testing
			Advanced Solid Tumors	Phase 1 NCT02792465	
			Breast Cancer	Phase 1 Phase 2 NCT05251714	
2	**BAY-1217389**		NA	Phase 1 NCT02366949	In vivo validation Clinical testing
3	**BOS172722**		Nonhaematologic Malignancies	Phase 1 NCT03328494	In vivo validation Clinical testing
			NA	Phase 1 NCT03464058	
4	**NMS-P715**		NA	NA	In vivo validation
5	**MPI-0479605**		NA	CHEMBL2047943	In vivo validation
6	**Reversine**		NA	CHEMBL188343	In vitro
7	**AZ3146**		NA	CHEMBL2140523	In vitro
8	**BAY-1161909**		NA	Phase 1 NCT02138812	In vivo validation, Clinical testing
9	**S-81694**	Structure undisclosed	Breast cancer	Phase 1 Phase 2 NCT03411161	In vivo validation, Clinical testing
10	**BAL-0891**		Refractory Acute Myeloid Leukemia	Phase 1 NCT05768932	Clinical testing

Within this framework, TTK functions as a buffering node that restrains chromosomal instability below its lethal threshold, allowing cancer cells to exploit the evolutionary advantages of CIN while maintaining sufficient mitotic fidelity for continued proliferation. Pharmacological inhibition of TTK does not simply inhibit a mitotic kinase; rather, it removes this buffering capacity. In tumors already operating with elevated baseline CIN, loss of TTK activity disrupts spindle assembly checkpoint signaling, resulting in premature anaphase onset, widespread chromosome mis-segregation, micronuclei formation, and progressive accumulation of aneuploidy that drives CIN beyond the tolerable threshold. In contrast, cells with lower baseline CIN, including most normal diploid cells, are generally less dependent on TTK-mediated buffering, providing a mechanistic basis for the therapeutic window observed in preclinical studies. Accordingly, TTK inhibitors exploit the CIN threshold by converting a level of chromosomal instability that is compatible with tumor evolution into one that is incompatible with tumor survival. This mechanistic framework provides the biological rationale for the TTK-targeted therapeutic strategies discussed below.

### Direct inhibitors targeting TTK

#### CFI-402257

CFI-402257 is a highly potent and selective ATP-competitive TTK inhibitor that has emerged as one of the most extensively characterized clinical candidates (enzyme IC₅₀ = 1.2 nM; Kᵢ = 0.09 nM) [115]. Inhibition of TTK by CFI-402257 abrogates SAC maintenance by disrupting kinetochore localization of MAD1/MAD2 complexes, thereby promoting premature anaphase onset and massive chromosomal segregation errors [104]. Importantly, preclinical studies demonstrated preferential cytotoxicity in CIN-high tumor models, while sparing normal diploid cells, highlighting a potential therapeutic window. Beyond mitotic catastrophe, recent evidence suggests that CFI-402257-induced micronucleation may activate cytosolic DNA sensing pathways, linking TTK inhibition to STING-mediated immune activation. These findings supported its progression into early-phase clinical evaluation and established it as a benchmark compound in the TTK inhibitor class [91].

#### BAY-1217389

BAY-1217389 is a selective TTK inhibitor developed for clinical translation with an enzyme IC_50_ below 10 nM [134]. Mechanistically, it enforces premature mitotic exit in the presence of spindle attachment errors, resulting in lethal chromosome missegregation [135]. Preclinical models demonstrated that combining BAY-1217389 with microtubule-stabilizing agents such as paclitaxel amplifies mitotic stress, suggesting a rational combinatorial strategy. Early-phase clinical studies further provided initial evidence supporting the feasibility of targeting TTK in solid tumors, strengthening the concept of mitotic checkpoint addiction as a therapeutic vulnerability [98].

#### BOS172722

BOS172722 is an orally bioavailable, selective TTK inhibitor optimized for improved pharmacokinetic and pharmacodynamic properties (IC₅₀ = 11 nM) [136]. Pharmacological inhibition leads to SAC collapse, multipolar spindle formation, micronucleation, and apoptotic cell death [137]. Preclinical studies revealed robust anti-tumor activity across multiple cancer models and synergistic interactions with taxanes. Phase I clinical evaluation demonstrated manageable toxicity profiles, supporting continued development of BOS172722 as a clinically relevant TTK-targeted agent [138].

#### NMS-P715

NMS-P715 was among the first selective small-molecule inhibitors developed against TTK and provided critical proof-of-concept for therapeutic checkpoint disruption. As an ATP-competitive inhibitor, NMS-P715 suppresses TTK kinase activity with an IC₅₀ of 182 nM in a standard assay, which decreases to approximately 8 nM following pre-incubation; its reported Kᵢ is approximately 0.99 nM [139], accelerates mitotic exit despite spindle defects, and induces widespread chromosome missegregation [140]. Preclinical studies showed preferential killing of chromosomally unstable cancer cells, thereby validating TTK as a druggable mitotic kinase. Although pharmacokinetic limitations prevented clinical advancement, NMS-P715 remains a foundational compound in mechanistic and preclinical studies of SAC biology [124].

#### MPI-0479605

MPI-0479605 is a potent and selective ATP-competitive inhibitor of TTK with an IC₅₀ of 1.8 nM [18] that promotes premature metaphase-to-anaphase transition and extensive mitotic abnormalities. Inhibition of TTK by MPI-0479605 results in aberrant chromosome segregation, mitotic catastrophe, and tumor growth suppression in preclinical models [141]. Similar to NMS-P715, this compound contributed significantly to early validation of TTK as a therapeutic target, although it did not progress to advanced clinical development [142].

#### Reversine

Reversine, originally identified as a dedifferentiation-inducing purine analogue, was later recognized as a dual inhibitor of TTK and Aurora kinases with reported TTK inhibitory potency in the low-nanomolar range. By inhibiting TTK, Reversine disrupts SAC enforcement and promotes chromosome missegregation, while concomitant Aurora kinase inhibition exacerbates mitotic spindle defects and cytokinesis failure. Although its lack of selectivity limits clinical applicability, Reversine provided early experimental evidence that simultaneous disruption of mitotic kinases can overwhelm tumor tolerance to CIN [121].

#### AZ3146

AZ3146 is a widely used ATP-competitive tool compound with a reported TTK IC₅₀ of approximately 35 nM that has been instrumental in defining the temporal requirement of TTK activity for checkpoint maintenance [143]. Acute inhibition of TTK by AZ3146 abolishes kinetochore recruitment of key checkpoint proteins, including MAD2 and BUBR1, leading to rapid SAC inactivation and mitotic slippage. While not developed for clinical use, AZ3146 has played a crucial role in elucidating the mechanistic consequences of acute checkpoint collapse and CIN induction [144].

#### BAY-1161909 (Empesertib)

BAY-1161909, also known as Empesertib, is a clinically evaluated selective TTK inhibitor designed to enforce mitotic checkpoint override. Preclinical studies demonstrated potent SAC suppression, induction of aneuploidy, and tumor regression in xenograft models [145]. Similar to other advanced inhibitors, BAY-1161909 has been explored in combination with microtubule-targeting agents to intensify mitotic stress and enhance tumor cell lethality, further supporting the translational relevance of TTK inhibition [135].

#### S-81694

S-81694 is a clinically evaluated TTK inhibitor with a reported IC₅₀ of approximately 3 nM [136]. Mechanistically, it disrupts SAC signaling, promotes premature mitotic exit, and induces chromosomal mis-segregation. Clinical findings reported disease stabilization in a subset of patients, providing additional evidence that pharmacological targeting of TTK is feasible and biologically active in human malignancies [146].

#### BAL-0891

BAL-0891 represents a next-generation dual TTK/PLK1 inhibitor designed to enhance mitotic disruption through coordinated targeting of two key mitotic regulators. Sustained TTK inhibition combined with transient PLK1 modulation results in rapid SAC collapse, aberrant mitotic exit, and profound chromosomal instability leading to cell death. This dual-target strategy may overcome compensatory resistance mechanisms and represents an emerging evolution in mitotic kinase targeting [147].

### Targeting mitotic surveillance pathways related to TTK

While TTK is the master initiator of SAC, its execution depends heavily on the recruitment of its associated kinases, including BUB1, Aurora B, and PLK1, which can be therapeutically targeted to indirectly disrupt TTK-dependent checkpoint signaling as summarized in Table 3.

**Table 3:** Indirect targeting of TTK: key mitotic kinase inhibitors affecting the spindle assembly checkpoint

Target	Inhibitor	Mode	Mechanism of Action	Reference
BUB1	BAY-1816032	ATP-competitive kinase inhibitor	Inhibits BUB1 catalytic activity and disrupts the H2A T120 phosphorylation pathway, leading to loss of SGO1 recruitment and displacement of the chromosomal passenger complex (CPC) from centromeres	[148]
	BAY-320	Small-molecule kinase inhibitor	Suppresses BUB1 kinase activity required for phosphorylation-dependent recruitment of checkpoint proteins at centromeres during mitosis	[149]
	BAY-524	Small-molecule inhibitor	Blocks BUB1-mediated histone phosphorylation at centromeres, interfering with the assembly of centromeric protein complexes necessary for chromosome segregation	[150]
	2OH-BNPP1	Chemical kinase inhibitor	Selectively inhibits engineered BUB1 kinase activity, preventing centromeric phosphorylation events involved in checkpoint signaling	[151]
Aurora B	Barasertib (AZD1152)	ATP-competitive Aurora B inhibitor	Inhibits Aurora B kinase activity, preventing phosphorylation events required for correction of improper kinetochore–microtubule attachments	[152]
	Tozasertib (VX-680)	Pan-Aurora kinase inhibitor	Blocks Aurora kinase catalytic activity, disrupting phosphorylation-dependent control of chromosome alignment and spindle checkpoint signaling	[153]
	ZM447439	Aurora kinase inhibitor	Suppresses Aurora B–dependent phosphorylation of kinetochore substrates involved in attachment error correction and chromosome segregation	[154]
PLK1	BI-2536	ATP-competitive PLK1 inhibitor	Inhibits PLK1 kinase activity required for centrosome maturation, spindle assembly, and kinetochore function during early mitosis	[155]
	Volasertib (BI-6727)	Selective PLK1 inhibitor	Blocks PLK1-dependent phosphorylation events controlling mitotic entry, spindle formation, and chromosome segregation	[156]

#### BUB1 inhibition

While TTK is the master initiator of SAC, its execution depends heavily on the recruitment of downstream kinases, most notably BUB1. Research led by Siemeister et al., 2019 demonstrates that BUB1 acts as a critical signal amplifier downstream of TTK. The development of BAY-1816032, a potent and selective ATP-competitive inhibitor of BUB1, has provided a tool to indirectly neutralize TTK-driven signalling [157]. By blocking BUB1’s catalytic activity, BAY-1816032 disrupts the H2ApT120/SGO1 axis, leading to the displacement of the CPC from the kinetochores [158]. Without a properly anchored CPC, the cell loses its ability to resolve attachment errors a process normally governed by TTK [159]. Beyond BAY-1816032, several other small molecules have been used to define the therapeutic landscape of BUB1 inhibition, which are BAY-320 [160], BAY-524 [161] and 2OH-BNPP1 [162].

#### Aurora kinase inhibitors

The functional interplay between TTK kinase and Aurora B kinase represents an attractive strategy for disrupting SAC signaling [163]. TTK initiates checkpoint signaling at unattached kinetochores, whereas Aurora B, the catalytic component of the CPC, ensures proper kinetochore-microtubule attachment by destabilizing erroneous spindle interactions [164]. These kinases operate within a tightly coordinated regulatory loop in which TTK activity promotes CPC recruitment, while Aurora B signaling helps maintain robust TTK localization at kinetochores [163]. Consequently, simultaneous inhibition of both kinases can collapse SAC signaling from two complementary directions. Pharmacological inhibition of Aurora B using agents such as Barasertib (AZD1152) [165], Tozasertib (VX-680) [166], and ZM447439 [167] disrupts error-correction mechanisms. When combined with TTK inhibition, the initiation of checkpoint signaling is also impaired, leading to premature mitotic exit, extensive chromosome mis-segregation, and ultimately mitotic catastrophe in rapidly proliferating cancer cells [168].

#### PLK1 Inhibitors

The mitotic kinase PLK1 plays a crucial role in kinetochore maturation [169], spindle assembly, and the recruitment of SAC proteins during early mitosis [170]. Functional interplay between PLK1 and TTK ensures proper checkpoint establishment and chromosome alignment [170]. Pharmacological inhibition of PLK1 using selective agents such as BI-2536 disrupts kinetochore organization and compromises the recruitment of several SAC components, thereby indirectly weakening TTK-dependent checkpoint signaling. As a consequence, PLK1 inhibition can trigger prolonged mitotic arrest followed by catastrophic mitotic exit, particularly in chromosomally unstable cancer cells [171]. These findings position PLK1 as a functionally relevant indirect target within the TTK signaling network and highlight the potential of combined PLK1–TTK targeting strategies to exacerbate mitotic stress and promote tumor cell death. Some other PLK inhibitors are Volasertib (BI-6727) [172], onvansertib [173], and GSK461364A [174].

#### Combination strategies involving TTK

TTK inhibition functions most effectively as a sensitizing strategy rather than a standalone cytotoxic approach. Given its role in enforcing SAC fidelity and allowing tumor cells to tolerate CIN, pharmacological inhibition of TTK renders cancer cells highly vulnerable to therapies that induce mitotic stress or DNA damage. Accordingly, rational combination strategies have been explored to enhance therapeutic efficacy.

#### TTK Inhibitors combined with microtubule-targeting agents

Microtubule-targeting agents, such as paclitaxel, disrupt spindle dynamics, leading to SAC activation and mitotic arrest. CIN-high tumor cells often adapt to this arrest and survive via mitotic slippage. Inhibition of TTK abrogates SAC enforcement, forcing premature mitotic exit despite persistent spindle defects and leading to extensive chromosome mis-segregation and mitotic catastrophe.

This combination strategy is supported by strong preclinical evidence. Maia et al., 2018 demonstrated that MPS1 inhibitors synergize with low doses of taxanes to selectively eliminate aneuploid cancer cells both in vitro and in vivo. Functional characterization of the selective TTK inhibitor CFI-402257 further showed enhanced anti-tumor efficacy when combined with microtubule poisons in CIN-high tumor models. Additional studies in osteosarcoma and other solid tumors confirmed that MPS1 inhibition potentiates paclitaxel-induced cytotoxicity, reinforcing the generalizability of this approach. [168–170]. These findings have directly informed early-phase clinical programs evaluating Mps1.

#### Combination of TTK inhibition with radiotherapy

Radiotherapy induces dsDNA breaks that activate cell-cycle checkpoints, delaying mitotic entry and allowing DNA repair [175]. TTK inhibition compromises this protective response by impairing checkpoint recovery and homologous recombination (HR) repair, forcing cells to enter mitosis with unresolved DNA damage and resulting in catastrophic chromosome fragmentation [176]. Robust preclinical evidence supports the radio-sensitizing potential of TTK inhibition. Chandler et al., 2020 demonstrated that genetic and pharmacological inhibition of TTK radio-sensitized basal-like breast cancer models by suppressing HR repair, reducing RAD51 foci formation, and increasing persistent DNA damage, leading to reduced clonogenic survival and enhanced tumor control in vivo. Supporting evidence from studies on CFI-402257 further indicates that TTK inhibition amplifies radiation-induced genomic instability and mitotic failure. Collectively, these findings establish TTK as a therapeutically actionable vulnerability in cancer, where disruption of its activity compromises mitotic fidelity and amplifies genomic instability beyond tolerable thresholds. While ATP-competitive inhibitors have demonstrated promising preclinical and early clinical efficacy, emerging strategies suggest that effective targeting of TTK may require multi-modal approaches that extend beyond kinase inhibition alone. Given its central role in integrating mitotic checkpoint signaling with stress-adaptive survival pathways, including the DNA damage response and apoptotic regulation, TTK inhibition is particularly well-suited for combination strategies that exploit mitotic and replication stress and therapeutic resistance mechanisms. Future therapeutic development will likely benefit from integrating pharmacological inhibitors with genetic and epigenetic targeting approaches to achieve more selective and durable anti-tumor responses.

### Mechanisms of resistance to TTK Inhibition

As TTK inhibitors advance toward clinical application, understanding the mechanisms underlying therapeutic resistance has become increasingly important. Current evidence indicates that resistance is driven by multiple mechanisms, including target-site alterations, adaptive mitotic responses, and pharmacological mechanisms that collectively reduce the effectiveness of TTK inhibition and limit therapeutic efficacy. One of the best-characterized resistance mechanisms is the acquisition of target-site mutations within the TTK kinase domain. Recurrent mutations, including I531M, I598F, C604Y, and S611R, reduce inhibitor binding while preserving TTK catalytic activity, thereby preserving TTK activity and downstream spindle assembly checkpoint (SAC) signaling despite inhibitor treatment. These mutations also exhibit variable sensitivity to structurally distinct inhibitors, suggesting that alternative binding modes may overcome mutation-mediated resistance [30]. Structural analyses further showed that conformational changes involving the P-loop, αC-helix, and activation loop decrease inhibitor affinity, providing a molecular framework for the rational design of next-generation TTK inhibitors [177].

While target-site mutations directly compromise inhibitor binding, not all resistant tumors acquire genetic alterations in TTK. Cancer cells can also acquire resistance through adaptive remodeling of mitotic checkpoint regulation, thereby reducing their functional dependence on TTK-mediated signaling. Genome-wide CRISPR loss-of-function screening identified multiple components of the APC/C as key determinants of resistance to CFI-402257. APC/C impairment delayed mitotic progression, providing additional time for chromosome attachment and enabling cells to survive persistent chromosome missegregation, thereby increasing tolerance to CIN despite TTK inhibition [178]. Consequently, rather than progressing to mitotic catastrophe, these cells may undergo mitotic slippage and re-enter interphase as highly aneuploid or polyploid cells with continued proliferative capacity. In addition, activation of complementary mitotic regulators, including Aurora kinases and PLK1, has been proposed to compensate for TTK inhibition and sustain mitotic progression, further promoting therapeutic resistance [179]. In addition to adaptive mitotic responses, resistance may also arise through pharmacological mechanisms that limit intracellular drug exposure. Genome-wide CRISPR activation screening identified ABCG2 as the dominant resistance-conferring gene for CFI-402257. ABCG2 overexpression reduced intracellular drug accumulation and attenuated therapeutic response, whereas its genetic or pharmacological inhibition restored drug sensitivity, supporting ABCG2-mediated drug efflux as a mechanism of resistance [180]. Collectively, resistance to TTK inhibition is a multifaceted process involving target-site mutations, reduced reliance on TTK-mediated checkpoint signaling, compensatory mitotic signaling, altered CIN tolerance, mitotic slippage, and ABCG2-mediated drug efflux. A comprehensive understanding of this resistance mechanisms will be essential for guiding the development of next-generation TTK inhibitors and rational combination therapies aimed at improving therapeutic efficacy and overcoming acquired resistance.

## Discussion

TTK plays a dual and context-dependent role in cancer, functioning both as a guardian of genomic stability and as a facilitator of tumor cell survival. Rather than acting solely as a component of the SAC, TTK emerges as a central regulatory node that links mitotic control with cellular responses to genotoxic and replication stress [181]. Within this framework, TTK serves as a key determinant of tumor cell fitness [182]. Its upregulation across multiple cancer types is consistently associated with aggressive tumor phenotypes and poor clinical outcomes [183]. Although elevated TTK expression may serve as a marker of proliferative or mitotically active states and is associated with adverse tumor phenotypes, functional dependency should be inferred only when genetic or pharmacological evidence demonstrates a requirement for TTK activity. Collectively, available evidence demonstrates strong correlations between elevated TTK expression and tumor progression, while functional disruption of TTK leads to reduced cellular viability, mitotic defects [184], and apoptosis [116] in diverse cancer models. In addition, TTK activity has been linked to EMT [68] and invasive behavior, further underscoring its contribution to tumor progression [185]. Despite this clear biological relevance, the clinical application of TTK as a biomarker remains limited. Variability in expression across tumor subtypes and the absence of standardized thresholds for defining “TTK-high” tumors represent major challenges. These limitations highlight the need for improved biomarker development and patient stratification strategies to enable more effective clinical translation.

Beyond its canonical mitotic role, TTK participates in a range of non-canonical signaling pathways. Mechanistic evidence places TTK at the interface of DDR and replication stress signaling, where it contributes to checkpoint activation and genome surveillance. Its regulation of CHK2 activation [86] and RPA2 phosphorylation [62] supports a functional role in coordinating DNA damage signaling with replication stress responses. In addition to these stress-associated pathways, TTK engages oncogenic signaling networks, including AKT–mTOR signaling, thereby promoting proliferation, metastasis, and tumor progression. Its involvement in EMT further links mitotic checkpoint dysregulation to cellular plasticity and invasive potential [186]. Transcriptomic and single-cell RNA sequencing analyses extend these observations by revealing that TTK expression is heterogeneously distributed across tumor cell populations. This heterogeneity associates TTK activity with proliferative, stress-adaptive, and plasticity-driven cellular states, suggesting a role in shaping intratumoral heterogeneity and facilitating dynamic tumor evolution [187, 188].

From a therapeutic perspective, TTK represents a promising target due to its essential role in maintaining mitotic fidelity in cancer cells. Preclinical studies consistently demonstrate potent antitumor activity of TTK inhibitors, including CFI-402257 [189] and BOS172722 [190]. Early clinical investigation of inhibitors such as BAY-1217389 further support the feasibility of targeting TTK [191]. However, several challenges continue to limit clinical translation. As mitotic kinases are also required for normal cell division, systemic inhibition of TTK carries a risk of toxicity in proliferating healthy tissues [110]. Moreover, tumors may activate compensatory signaling pathways or adapt their level of CIN, thereby reducing dependence on TTK and limiting the efficacy of monotherapy.

Combination strategies offer a compelling approach to overcoming these limitations. Inhibition of TTK has been shown to sensitize tumor cells to microtubule-targeting agents and radiotherapy [192], with synergistic effects observed in combination with taxanes and enhanced radiosensitivity linked to impaired DNA repair [193]. These findings support the integration of TTK inhibitors into combination regimens designed to exploit mitotic and genotoxic stress. Despite significant advances, several key questions remain unresolved. The spatial and temporal regulation of TTK activity, particularly the coordination between its mitotic and non-mitotic functions, is not fully understood. It also remains unclear whether these functions can be selectively targeted or whether they represent interconnected aspects of a unified regulatory system. Furthermore, identifying predictive biomarkers of TTK inhibitor sensitivity remains a major unmet need.

Collectively, TTK operates at the intersection of mitotic surveillance, genome maintenance, and tumor adaptation. By integrating checkpoint signaling with broader stress-response pathways, it enables cancer cells to tolerate genomic instability while sustaining proliferation. Targeting TTK-dependent mitotic stress adaptation, therefore, represents a promising strategy to selectively eliminate chromosomally unstable cancers, if challenges related to specificity, resistance, and patient stratification can be effectively addressed.

## Conclusion

TTK has emerged as a central regulator of mitotic fidelity, extending far beyond its classical role in the SAC. As highlighted throughout this review, TTK integrates kinetochore signaling with broader cellular stress-response pathways, including the DDR, replication stress signaling, and apoptotic regulation. This functional expansion positions TTK not merely as a mitotic kinase but as a critical mediator of tumor adaptation. A unifying concept that emerges is that cancer cells exploit TTK to tolerate elevated levels of CIN, thereby sustaining proliferation under conditions that would otherwise be lethal. In this context, TTK functions as a buffering node that enables survival despite ongoing genomic imbalance. Therapeutically, this creates a unique vulnerability: inhibition of TTK does not simply disrupt mitosis but drives tumor cells beyond the threshold of tolerable CIN, resulting in catastrophic genome missegregation and cell death. Despite promising preclinical and early clinical advances, several challenges remain. Identifying robust predictive biomarkers of TTK inhibitor sensitivity is essential, as CIN alone may not fully define therapeutic response. In addition, resistance mechanisms and the potential toxicity associated with targeting essential mitotic processes must be carefully addressed. Future strategies will likely require rational combination approaches that integrate TTK inhibition with therapies inducing mitotic or genotoxic stress. Moving forward, a deeper understanding of how TTK coordinates its canonical and non-canonical functions will be critical. In particular, dissecting the context-specific dependencies of tumor cells on TTK activity may enable more selective and effective therapeutic targeting. Overall, TTK represents a compelling example of how mitotic regulators are co-opted during tumor evolution, and targeting TTK-dependent stress adaptation may provide a promising avenue for the treatment of chromosomally unstable cancers.

## Data Availability

No datasets were generated or analysed during the current study.

## Abbreviation

TTK: Tyrosine Threonine Kinase
MPS1: Monopolar Spindle 1
SAC: Spindle Assembly Checkpoint
CIN: Chromosomal Instability
DDR: DNA Damage Response
MCC: Mitotic Checkpoint Complex
APC/C: Anaphase Promoting Complex/Cyclosome
CDC20: Cell Division Cycle 20
KNL1: Kinetochore Null Protein 1
BUB1: Budding Uninhibited by Benzimidazoles 1
BUBR1: BUB1 Related Kinase
MAD1: Mitotic Arrest Deficient 1
MAD2: Mitotic Arrest Deficient 2
CHK2: Checkpoint Kinase 2
ATM: Ataxia Telangiectasia Mutated
ATR: Ataxia Telangiectasia and Rad3 Related
RPA: Replication Protein A
EMT: Epithelial–Mesenchymal Transition
PKCα: Protein Kinase C Alpha
ERK: Extracellular Signal-Regulated Kinase
MAPK: Mitogen-Activated Protein Kinase
STAT3: Signal Transducer and Activator of Transcription 3
AKT: Protein Kinase B
mTOR: Mechanistic Target of Rapamycin
CPC: Chromosomal Passenger Complex
PLK1: Polo-Like Kinase 1
TNBC: Triple-Negative Breast Cancer
NSCLC: Non-Small Cell Lung Cancer
HCC: Hepatocellular Carcinoma
CRC: Colorectal Cancer
HR: Homologous Recombination
DSB: Double-Strand Break
BER: Base Excision Repair
Ndc80: Nuclear Division Cycle 80 complex
CDK1: Cyclin-Dependent Kinase 1
CPC: Chromosomal Passenger Complex
SPB: Spindle Pole Body
TPR: Tetratricopeptide Repeat
NTE: N-Terminal Extension
MDS: Middle Domain Segment
C-MAD2: Closed conformation of MAD2
O-MAD2: Open conformation of MAD2
H2ApT120: Histone H2A phosphorylated at threonine 120
SGO1: Shugoshin 1
EMT: Epithelial–Mesenchymal Transition
NF-κB: Nuclear Factor Kappa-B
Akt: Protein Kinase B
mTOR: Mechanistic Target of Rapamycin
NMS-P715: MPS1 kinase inhibitor
OSU-13: TTK/MPS1 inhibitor
BI-2536: Polo-like kinase 1 inhibitor
AZD1152: Aurora kinase inhibitor (Barasertib)
VX-680: Aurora kinase inhibitor (Tozasertib)
ZM447439: Aurora kinase inhibitor
